# Clinical and Pathological Features That Predict High-Grade B-Cell Lymphomas (HGBCLs) with *MYC* and *BCL2* or *BCL6* Translocations (Double-Hit Lymphoma): A Systematic Review and Meta-Analysis

**DOI:** 10.3390/biomedicines14061375

**Published:** 2026-06-18

**Authors:** Ernest Lee, Wai Ying Katherine Wong, Han-Chieh Yang, Elizabeth J. Soilleux

**Affiliations:** 1 School of Clinical Medicine, University of Cambridge, Cambridge CB2 0SP, UK; 2 Department of Cellular Pathology, St Thomas’ Hospital, London SE1 7EH, UK; 3 Cancer Epidemiology Unit, Nuffield Department of Population Health, University of Oxford, Oxford OX3 7LF, UK; 4 Department of Pathology, University of Cambridge, Cambridge CB2 0SP, UK; 5 Haematopathology and Oncology Diagnostic Service (HODS), Cambridge University Hospitals NHS Foundation Trust, Cambridge CB2 0QQ, UK

**Keywords:** double-hit lymphoma, diffuse large B-cell lymphoma, high-grade B-cell lymphoma, immunophenotype, clinical, morphology, predictive features, FISH, *MYC/BCL2/BCL6*, translocations

## Abstract

**Background/Objectives:** High-grade B-cell lymphomas (HGBCLs) with *MYC* and *BCL2* or *BCL6* translocations, colloquially referred to as double-hit lymphomas (and abbreviated here to DHL), are aggressive malignancies. Differentiating DHL from non-DHL HGBCLs is important, as DHL patients may benefit from more intensive treatment regimes. We aimed to identify predictive clinicopathological, morphological, and immunophenotypic features that could guide selection of HGBCLs for fluorescence in situ hybridization (FISH), which is expensive and less accessible in some centers. **Methods:** We conducted a PRISMA systematic review and meta-analysis on 29 studies identified from four databases (PubMed (MEDLINE), Ovid (Embase), Web of Science, and Scopus). We calculated risk ratios (RRs) to compare features between DHL and non-DHL HGBCL and between *MYC/BCL2* and *MYC/BCL6* DHL patients. **Results:** DHL patients were associated with higher Ann Arbor stage (RR 1.15, *p* = 0.028, *I*^2^ = 38.7%), International Prognostic Index (IPI) score (RR 1.27, *p* = 0.047, *I*^2^ = 37.9%), elevated lactate dehydrogenase (RR 1.26, *p* = 0.012, *I*^2^ = 34.0%), and germinal center B-cell-like (GCB) immunophenotype (RR 1.21, *p* = 0.043, *I*^2^ = 35.8%) compared to non-DHL HGBCL patients. c-Myc immunopositivity, extranodal disease, and bone marrow involvement were more likely in DHL, albeit not reaching statistical significance. Extranodal disease (*p* = 0.015, *I*^2^ = 0.0%), central nervous system involvement (*p* = 0.044, *I*^2^ = 0.0%), and non-GCB immunophenotype (*p* = 0.016, *I*^2^ = 71.1%) were more likely in *MYC/BCL6* compared to *MYC/BCL2* DHL patients. BCL2 immunopositivity, CD10 immunopositivity, and MUM1 immunonegativity were more likely in *MYC/BCL2* DHL, although the differences were not statistically significant. **Conclusions:** Our results have associated DHL with features of aggressive disease and found GCB immunophenotype as a histopathological feature with statistically significant predictive value for MYC*/BCL2* DHL. Heterogeneity within the non-DHL HGBCL group and variation in immunohistochemical cut-off values between studies limited identification of other predictive features. Larger, consistently designed, prospective cohort studies could provide further evidence for a screening strategy for DHL.

## 1. Introduction

High-grade B-cell lymphoma (HGBCL) harboring *MYC* (8q24.2) and *BCL2* (18q21.3) and/or *BCL6* (3q27.3) rearrangement, also known colloquially as “double-hit lymphoma” (DHL), was introduced in the 2016 4th edition of the World Health Organization (WHO) Classification of Haematolymphoid Tumors (WHO-HAEM4) [1]. These entities account for 5–10% of all HGBCL, with *MYC/BCL2* being the most common type (65–72% and 0.3–0.8 cases per 100,000 person-years), followed by *MYC/BCL6* (10–14%) [2,3,4,5,6].

HGBCL with *MYC/BCL6* rearrangements have since been removed from a specific “double-hit” category in the 2022 5th Edition WHO-HAEM5, recognizing that these malignancies have more heterogeneous gene expression profiles and mutational landscapes compared to *MYC/BCL2-*rearranged HGBCL [7]. These malignancies more commonly harbor mutations such as PIM1 and CD79B, linking them to the Activated B-Cell-like (ABC)-diffuse large B-cell lymphoma (DLBCL) subtype, whereas *MYC/BCL2*-rearranged HGBCLs possess more frequent epigenetic regulator gene mutations and cluster with follicular lymphoma and EZB-DLBCL [7].

In WHO-HAEM5, *MYC/BCL6*-rearranged HGBCL have now been reclassified as genetic subtypes of DLBCL, not otherwise specified (NOS), or of HGBCL, NOS [7]. The differentiation between these two groups is based on differences in morphology, with the former characterized by medium-sized to large B cells with a diffuse growth pattern that do not meet diagnostic criteria for specific large B-cell lymphomas, and the latter by medium-sized or blastoid cells that do not meet the diagnostic criteria for other defined lymphoma entities [7]. *MYC/BCL6-*rearranged HGBCL remains a provisional entity within the “double-hit HGBCL” category according to the 2022 International Consensus Classification [8]. Consistent with this, the WHO-HAEM5 classification continues to recommend reporting *MYC/BCL6*-rearranged cases to facilitate patient recruitment into research studies, given the incomplete biological and clinical characterization of these neoplasms [7].

Differentiating between DHL and non-DHL HGBCL (abbreviated here as non-DHL) is crucial, as DHL patients (possessing either *MYC*/*BCL2* or *MYC*/*BCL6* rearrangements) often present with a more aggressive clinical course and are known to have poorer prognosis when compared to, for example, single-hit DLBCL, which have *MYC* or *BCL2* rearrangements alone or other molecular aberrations [9,10,11,12]. As a result, DHL patients often require more intensive chemoimmunotherapy than the conventional rituximab, cyclophosphamide, doxorubicin, vincristine, and prednisone (R-CHOP) regimen [13]. These include a dose-adjusted regimen of etoposide, prednisone, vincristine, cyclophosphamide, and doxorubicin (DA-EPOCH), hyperfractionated cyclophosphamide, vincristine, doxorubicin, and dexamethasone (Hyper-CVAD), and cyclophosphamide, vincristine, doxorubicin, and cytarabine with methotrexate/etoposide, ifosfamide, and cytarabine (CODOX-M/IVAC) [13].

Accurate and timely diagnosis of *MYC/BCL2-* or *MYC/BCL6*-rearranged DHL, by identification of *MYC*, *BCL2* or *BCL6* gene rearrangement, is imperative to guide optimal treatment decisions. Fluorescence in situ hybridization (FISH) for *MYC*, *BCL2* and *BCL6* translocations is the current “gold standard” test in clinical practice for detecting the presence of these translocations. The WHO-HAEM5 guidelines advise a sequential strategy starting with evaluation of *MYC* translocation followed by the evaluation of *BCL2* (and *BCL6*) rearrangement status and *MYC* translocation partner [7]. Targeted next-generation sequencing techniques have been suggested as the next step of screening to more precisely identify genetic alterations (such as single-nucleotide variants or copy number variations), as well as *MYC* translocation partners, which have clinical prognostic implications and may not be easily identifiable with routine interphase FISH [14,15].

There are currently no universally agreed-upon strategies or guidelines for selecting which HGBCLs should undergo FISH testing [7]. Proposed immunophenotypic strategies to identify DHL include germinal center B-cell-like (GCB) cell-of-origin (COO) immunohistochemistry [4,16], GCB COO immunohistochemistry testing together with c-Myc and BCL2 dual expression [4], and moderate to strong c-Myc immunostaining in >70% of neoplastic cells [17,18,19]. While pragmatic and cost-saving, these approaches have limitations, including missing a proportion of DHL cases and lacking specificity for identifying DHL. For example, some HGBCLs with high c-Myc expression do not carry *MYC* translocations [19].

The lack of reliable strategies may be attributable to overlapping biological features between DHL and non-DHL cases, as well as between the *MYC/BCL2* and *MYC/BCL6-*rearranged DHL (abbreviated here to *MYC/BCL2* and *MYC/BCL6* DHL, respectively) [12]. Furthermore, our knowledge of DHL comes predominantly from studies with small sample sizes reflecting the relative underutilization, until recently, of FISH for diagnosing DHL, resulting in its under-diagnosis [20]. Without reliable stratification strategies, many centers have resorted to routine FISH screening strategies for all HGBCL cases. However, testing is typically performed in centralized cancer centers with turnaround time ranging from days to weeks [21]. Access in more resource-limited settings may be limited, delaying treatment decisions [20,22].

In this review, we aimed to collate results from multiple studies to identify predictive clinical and histopathological features to screen potential DHL patients for FISH testing.

## 2. Materials and Methods

### 2.1. Search Strategy

This systematic review was conducted in accordance with the Preferred Reporting Items for Systematic Reviews and Meta-Analysis (PRISMA) guidelines (Supplementary Table S1) [23]. A literature search was performed from conception to July 2025 using the following databases [24]: (1) PubMed (MEDLINE), (2) Ovid (Embase), (3) Web of Science, and (4) Scopus. The search strategy is shown in Table 1. This review was registered in PROSPERO (CRD42021269567) [25] and included up-to-date studies until July 2025. A manual bibliography search was not performed. All studies identified by the search were imported into the Rayyan website and deduplicated [26]. Title, abstract, and full-text screening were independently performed by E.L. and W.Y.K.W. Disagreements were resolved by consensus or through discussion with a third reviewer (E.J.S.).

### 2.2. Inclusion and Exclusion Criteria

We included eligible studies available in full-text English with clinicopathological, morphological, immunophenotypic, and cytogenetic features of DHL patients. Corresponding data from a comparison group, defined as any large B-cell lymphomas or HGBCL without double-hit (DH) translocations, were also extracted. The inclusion and exclusion criteria are shown in Table 2 using the Population, Intervention, Comparison, Outcome, Study type (PICOS) model as a guide [27].

### 2.3. Data Extraction

Data extraction was independently performed by E.L. and W.Y.K.W. A data extraction strategy was agreed upon amongst reviewers prior to data extraction. Discrepancies were resolved by consensus or through discussion with a third reviewer (E.J.S.). A template of the data extraction form is provided in Supplementary Table S2. Data were extracted from each included study onto an Excel spreadsheet, when available, including:

1. Study characteristics and subject demographics.
2. Clinical features: Age, Sex, Ann Arbor stage, International Prognostic Index (IPI) score, serum lactate dehydrogenase (LDH) level, Eastern Cooperative Oncology Group (ECOG) performance status (PS), extranodal involvement and affected sites, and the presence of B symptoms, BM, and CNS involvement.
3. Histopathological features: immunophenotype (immunohistochemistry and flow cytometry), cell-of-origin (COO) (germinal center B-cell (GCB) versus non-germinal center B-cell-like (non-GCB)), and morphology.
4. Cytogenetic and molecular features: fluorescent in situ hybridization (FISH) and Epstein–Barr virus (EBV) status.

### 2.4. Statistical Analyses

Meta-analyses were conducted using RStudio version 5.5.1. For binary outcomes, a random-effects model for risk ratios (RR), between DHL and non-DHL cases and between *MYC/BCL2* and *MYC/BCL6* DHL, was implemented using the Mantel-Haenszel method within the metabin package [28]. The Paule-Mandel estimator for tau-squared, and Higgins and Thompson’s I-squared statistic were used to assess heterogeneity [29]. Pooled proportion effect sizes were calculated with the metaprop package [28] from logit transformed values of the raw proportions, which were used to build a generalized linear mixed-effects model. *p*-values < 0.05 were considered to be statistically significant. Forest plots were used for graphic presentation of meta-analyzed results. Comparison between RR and pooled proportions served as a sensitivity analysis. An available-case analysis approach was adopted for handling missing data from studies [30].

There is debate on when and how adjustments for multiple testing should be performed, with some viewing that exploratory analyses should be performed without multiplicity adjustment [31,32]. In this meta-analysis, we compared pre-defined clinicopathological features between DHL and non-DHL groups. These features had been pre-selected based on their previously reported use as proposed pre-screening features for FISH testing and constitute distinct clinicopathological features. Furthermore, each DHL versus non-DHL comparison was performed on different subsets of patients across the studies, reflecting variability in the reporting of clinicopathological features. Given these considerations, adjustment for multiple testing was not performed in this study.

The Tau-squared statistic was used to estimate between-study heterogeneity, calculated with the maximum-likelihood estimator. Similarly, *I*^2^ values were calculated to measure effect size variance across studies, interpreted as low (0–25%), moderate (25–50%), substantial (50–75%), and critical (>75%) [29]. Meta-analyses were only conducted when at least three studies were available, with each study contributing at least three or more observations. For the comparison between *MYC/BCL2*- and *MYC/BCL6* DHL, the latter was used as the control group. Non-meta-analyzed parameters were analyzed using descriptive statistics.

Publication bias was determined by the degree of symmetry of funnel plots and Egger’s test, on parameters with greater than or equal to ten studies.

### 2.5. Risk of Bias Assessment

Quality assessment was performed independently by E.L. and W.Y.K.W. using the National Institute of Health/National Heart, Lung, and Blood Institute (NIH/NHLBI) Quality Assessment Tool for Observational Cohort and Cross-Sectional Studies and Case Series Studies. Disagreements were resolved by consensus or through discussion with a third reviewer (EJS). The summary of the overall results of the risk of bias assessment is presented in Supplementary Tables S3 and S4.

## 3. Results

### 3.1. Study Selection and Characteristics

**Figure 1:**
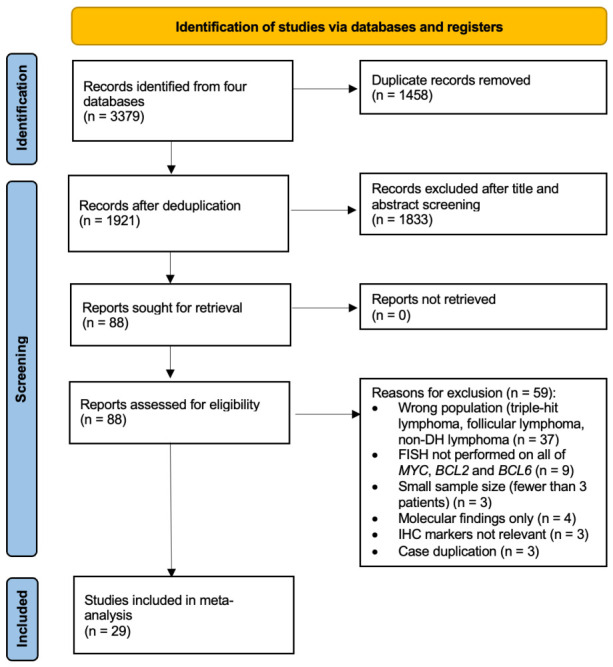
PRISMA flow-chart of article screening and selection.

A total of 3379 studies were identified from the literature search, with 1921 remaining after deduplication for title and abstract screening (Figure 1). Eighty-eight studies were selected for full-text screening based on the inclusion and exclusion criteria. Twenty-eight retrospective studies and one prospective non-randomized study were included (Table 3) [11,33,34,35,36,37,38,39,40,41,42,43,44,45,46,47,48,49,50,51,52,53,54,55,56,57,58,59,60]. A total of 773 DHL cases, of which 471 and 246 were classified as *MYC/BCL2* and *MYC/BCL6* DHL, respectively, were included. The median age of DHL patients ranged from 48.0 to 72.3 years. Males accounted for 14.3–100% of cases. Fourteen studies included comparative non-DHL groups, totaling 1237 patients.

### 3.2. Clinical Features

**Figure 2:**
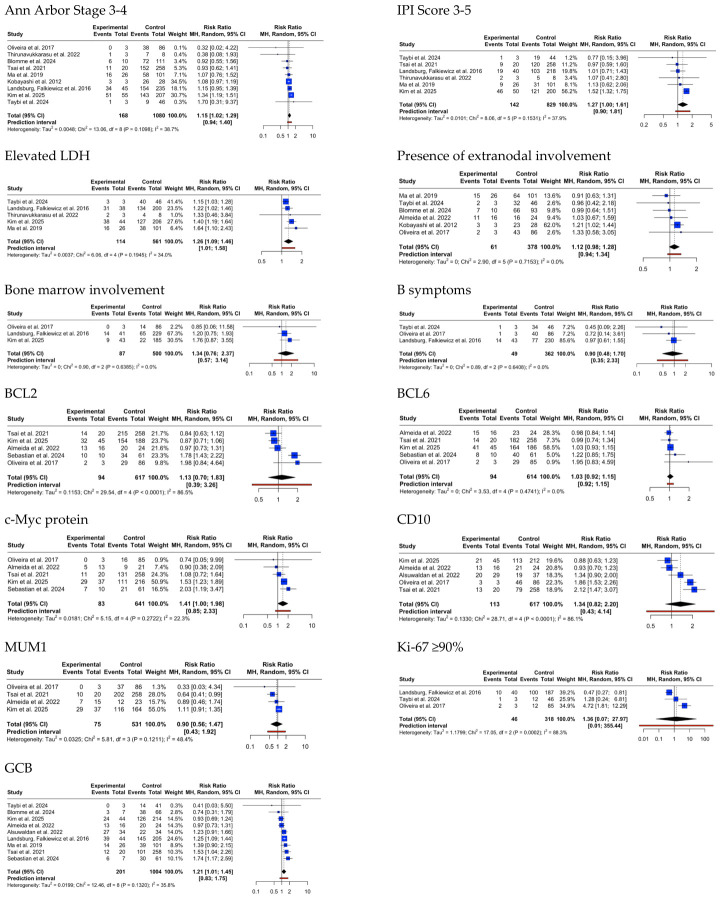
Forest plots of pooled analysis comparing the risk ratios (RRs) of clinicopathological features between DHL and non-DHL cases [11,34,43,46,50,52,53,55,56,58,59,60].

Compared to non-DHL patients, DHL patients were more likely to exhibit higher-stage disease (RR 1.15, *p* = 0.028), higher IPI scores (RR 1.27, *p* = 0.047), and elevated LDH levels (RR 1.26, *p* = 0.012) (Figure 2). The presence of extranodal disease (RR 1.12, *p* = 0.078) and BM involvement (RR 1.34, *p* = 0.16) was also more likely in DHL patients, although these associations were not statistically significant. There was no significant difference between the presence of B symptoms between DHL and non-DHL patients (RR 0.90, *p* = 0.55).

Pooled proportions of clinical features amongst DHL patients are shown in Table 4. Across eleven studies (374 patients), the pooled proportion of DHL patients with bone marrow (BM) involvement was 38.8%. Central nervous system (CNS) involvement in DHL patients, pooled from ten studies, was 14.8%. Disease localization was reported in seven studies, one of which included cases that all had oropharyngeal involvement [49]. The most common presenting sites were lymph nodes (26.5%), followed by the gastrointestinal tract (13.6%). Meta-analysis of CNS involvement was not performed between DHL and non-DHL due to insufficient studies reporting this in both groups.

**Figure 3:**
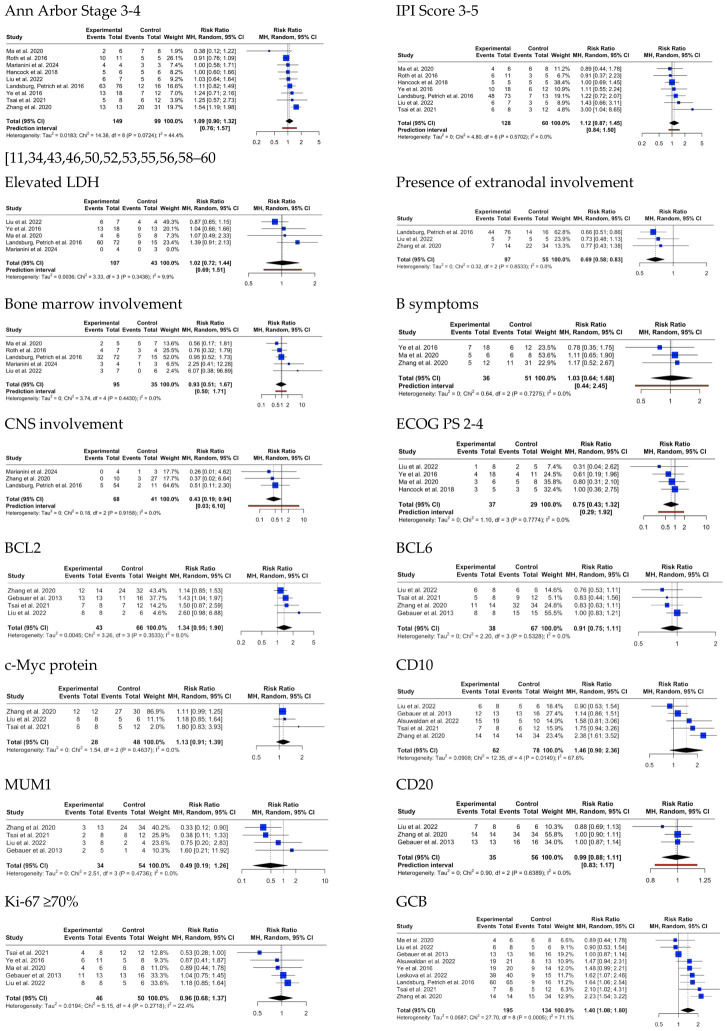
Forest plots of pooled analysis comparing the risk ratios (RRs) of clinicopathological features between *MYC/BCL2* and *MYC/BCL6* DHL cases [36,38,39,41,45,47,48,50,51,53,54,57].

Compared to *MYC/BCL6* DHL patients, *MYC/BCL2* DHL patients were less likely to have extranodal disease (RR 0.69, *p* = 0.013) and CNS involvement (RR 0.43, *p* = 0.044). ECOG PS was lower in *MYC/BCL2* than *MYC/BCL6* patients, although not reaching statistical significance (RR 0.75, *p* = 0.21). There was no significant difference in disease staging, IPI score, the presence of LDH elevation, BM involvement, and B symptoms (Figure 3). Pooled proportions of clinical features amongst *MYC/BCL2* and *MYC/BCL6* patients are shown in Table 5 and Table 6.

### 3.3. Histopathological Features

c-Myc protein expression was more likely in DHL than non-DHL patients (RR 1.41, *p* = 0.051) and approached but did not reach statistical significance. There were also no significant differences in BCL2, BCL6, CD10, and MUM1/IRF4 immunostaining (Figure 2). Based on the Hans classification for cell-of-origin (COO) determination, GCB phenotype was significantly more likely in DHL patients (RR 1.21, *p* = 0.043). Pooled proportions of immunohistochemical expression and COO of DHL patients are shown in Table 4. Proportions of other IHC (CD19, PAX5, CD79a, TdT) and flow cytometry markers (CD19, CD20, CD10, CD45, CD34, TdT, and light chain) are shown in Supplementary Tables S5 and S6.

Compared to *MYC/BCL6*, *MYC/BCL2* DHL patients were more likely to be of GCB phenotype (RR 1.40, *p* = 0.016) (Figure 3). CD10 immunopositivity (RR 1.46, *p* = 0.098) and MUM1 immunopositivity (RR 0.49, *p* = 0.095) were more likely in *MYC/BCL2* DHL patients although not reaching statistical significance. BCL6 negative (RR 0.91, *p* = 0.23) and BCL2 positive (RR 1.34, *p* = 0.071) immunostaining was more likely in *MYC/BCL2* DHL patients but this difference was not statistically significant. Expression of c-Myc protein and CD20 also did not differ between the two subtypes (Figure 3).

Three of the five studies that evaluated Ki-67 proliferation index between DHL and non-DHL patients defined high Ki-67 expression as ≥90% [11,43,58], one used ≥80% [46] and the other ≥70% [50] (Table 7). No significant difference in Ki-67 expression levels between the two groups was observed. Out of the six papers which compared Ki-67 in the *MYC/BCL2* versus *MYC/BCL6* groups, three studies used a cut-off of ≥70% [38,48,50], one study used a cut of ≥90% [39], and two studies reported individual Ki-67 values [36,54] (Table 7). As most studies used a ≥70% Ki-67 positivity threshold or reported individual Ki-67 values to which this threshold could be applied, ≥70% was used as the positivity cut-off value for the *MYC/BCL2* versus *MYC/BCL6* Ki-67 comparison, with no significant difference observed between these two groups.

DHL morphology was reported in 16 studies. Seven studies which preselected DHL based on morphology were excluded from this analysis. The remaining nine studies comprised 252 patient samples, which had undergone FISH testing to identify DHL cases and were subsequently evaluated for morphology. We recognize that the morphological subtypes of HGBL are poorly defined and demonstrate limited reproducibility. “DLBCL NOS” morphology describes morphologies that simply do not meet the diagnostic criteria for specific large B-cell lymphoma neoplasms [7]. Morphological classifications have also evolved over time. For example, “BCLU” introduced in the 2008 WHO classification, was removed in WHO-HAEM4, and reclassified as HGBCL, NOS, or HGBCL with *MYC* and *BCL2* and/or *BCL6* rearrangements [7]. Nonetheless “DLBCL” was the most common morphology (range 28.6–81.8%), followed by “BCLU” (range 0.0–66.7%) (Supplementary Table S7).

**Table 4:** Summary estimates (proportion as effect size, and pooled) of clinicopathological features of overall DHL cases. N /A = Not applicable.

	No. of Studies	No. of Cases	Proportion [95% CI] (%)	I^2^ Statistic [95% CI] (%)	τ^2^	Likelihood Ratio Test (LRT)	Egger’s Test *p*-Value
**Clinical features**							
Ann Arbor Stage 3-4	21	571	75.9 [68.1; 82.3]	45.8 [9.6; 67.5]	0.344	<0.0001	0.208
IPI Score 3-5	16	456	67.2 [54.5; 77.7]	69.9 [49.8; 82.0]	0.628	<0.0001	0.867
Elevated LDH	13	412	76.5 [65.2; 85.1]	3.5 [0.0; 58.1]	0.482	0.0004	0.402
Extranodal involvement	12	255	67.1 [60.3; 73.2]	0.0 [0.0; 58.3])	0	0.156	0.0071
Extranodal involvement ≥2 sites	6	310	43.7 [31.6; 56.6]	51.2 [0.0; 80.6]	0.0785	0.0556	N/A
BM involvement	11	374	38.8 [29.2; 49.4]	48.1 [0.0; 74.1]	0.168	0.0060	0.247
CNS involvement	10	250	14.8 [10.4; 20.6]	0.0 [0.0; 62.4]	0	0.160	0.814
B symptoms	6	136	43.0 [27.4; 60.1]	39.1 [0.0; 75.8]	0.1554	0.0751	N/A
ECOG PS 2-4	7	122	38.2 [19.8; 60.8]	52.1 [0.0; 79.6]	0.5355	0.0023	N/A
**Immunophenotypic features**							
BCL2	13	363	81.7 [74.3; 87.4]	29.6 [0.0; 63.6]	0.167	0.0340	0.725
BCL6	14	312	87.0 [80.0; 91.8]	0.0 [0.0; 55.0]	0.189	0.0248	0.330
c-Myc protein	10	215	76.6 [55.3; 89.7]	56.7 [12.2; 78.6]	1.32	<0.0001	0.990
CD20	6	113	97.4 [85.5; 99.6]	0.0 [0.0; 74.6]	0.591	0.170	N/A
CD10	14	395	81.2 [66.7; 90.3]	74.7 [57.3; 85.0]	1.156	<0.0001	0.0510
MUM1	11	172	51.8 [38.4; 64.9]	27.4 [0.0; 64.1]	0.258	0.0073	0.287
Ki-67 ≥70%	9	132	81.1 [69.5; 89.0]	1.8 [0.0; 65.4]	0.104	0.197	N/A
Ki-67 ≥90%	4	135	40.7 [18.2; 68.0]	64.0 [0.0; 87.8]	0.2173	0.028	N/A
GCB	19	495	75.4 [65.5; 83.3]	57.2 [28.6; 74.3]	0.631	<0.0001	0.719

**Table 5:** Summary estimates (proportion as effect size, and pooled) of clinicopathological features of *MYC/BCL2* DHL cases. N/A = Not applicable.

	No. of Studies	No. of Cases	Proportion [95% CI] (%)	I^2^ Statistic [95% CI] (%)	τ^2^	Likelihood Ratio Test (LRT)	Egger’s Test *p*-Value
**Clinical features**							
Ann Arbor Stage 3-4	14	324	80.8 [71.8; 87.4]	24.1 [0.0; 59.7]	0.153	0.0275	0.243
IPI Score 3-5	11	285	70.5 [59.9; 79.4]	32.4 [0.0; 66.8]	0.142	0.0466	0.505
Elevated LDH	9	257	80.9 [74.6; 86.0]	0.0 [0.0; 64.8]	0	0.0356	N/A
Extranodal involvement	5	122	60.7 [48.0; 72.1]	0.0 [0.0; 79.2]	0	0.616	N/A
Extranodal involvement ≥2 sites	4	194	39.6 [15.3; 70.4]	58.4 [0.0; 86.2]	0.202	0.0334	N/A
BM involvement	8	251	47.0 [39.7; 54.5]	0.0 [0.0; 67.6]	0	0.624	N/A
CNS involvement	6	161	10.9 [3.8; 27.6]	0.0 [0.0; 74.6]	0	0.105	N/A
B symptoms	3	36	47.2 [17.5; 79.0]	34.5 [0.0; 78.7]	0	0.133	N/A
ECOG PS 2-4	6	50	39.1 [17.2; 66.5]	0.0 [0.0; 74.6]	0.387	0.0386	N/A
**Immunophenotypic features**							
BCL2	11	240	90.0 [84.8; 93.6]	0.0 [0.0; 60.2]	0	0.182	0.319
BCL6	11	187	86.4 [77.4; 92.1]	0.0 [0.0; 60.2]	0.0882	0.208	0.601
MYC	8	122	89.8 [53.7; 98.5]	23.0 [0.0; 64.6]	2.6917	0.0003	N/A
CD20	4	46	95.7 [68.8; 100.0]	0.0 [0.0; 84.7]	0	0.2929	N/A
CD10	11	259	92.3 [83.7; 96.6]	28.3 [0.0; 64.7]	0.567	0.0289	0.996
MUM1	8	71	35.2 [23.2; 49.4]	0.0 [0.0; 67.6]	0	0.861	N/A
Ki-67 ≥70%	9	77	79.4 [62.3; 90.0]	0.0 [0.0; 64.8]	0.296	0.0936	N/A
GCB	14	380	93.7 [87.1; 97.0]	25.2 [0.0; 60.4]	0.8012	0.0059	0.5925

**Table 6:** Summary estimates (proportion as effect size, and pooled) of clinicopathological features of *MYC/BCL6* DHL cases. N/A = Not applicable.

	No. of Studies	No. of Cases	Proportion [95% CI] (%)	I^2^ Statistic [95% CI] (%)	τ^2^	Likelihood Ratio Test (LRT)	Egger’s Test *p*-Value
**Clinical features**							
Ann Arbor Stage 3-4	12	167	76.0 [61.9; 86.1]	39.7 [0.0; 69.5]	0.483	0.0039	0.496
IPI Score 3-5	9	113	65.3 [40.9; 83.7]	64.0 [26.3; 82.4]	1.043	<0.0001	N/A
Elevated LDH	7	90	72.7 [49.3; 88.0]	0.0 [0.0; 70.8]	0.542	0.0057	N/A
Extranodal involvement	5	68	74.7 [50.3; 89.6]	0.0 [0.0; 79.2]	0.0624	0.174	N/A
Extranodal involvement ≥2 sites	3	70	48.6 [25.2; 72.6]	0.0 [0.0; 89.6]	0	0.593	N/A
BM involvement	6	78	37.3 [14.2; 68.1]	50.2 [0.0; 80.2]	0.827	0.006	N/A
CNS involvement	5	76	17.1 [8.1; 32.5]	0.0 [0.0; 79.2]	0	0.780	N/A
B symptoms	4	54	45.1 [20.3; 72.7]	23.2 [0.0; 88.2]	0.029	0.220	N/A
ECOG PS 2-4	4	29	48.3 [22.2; 75.3]	0.0 [0.0; 84.7]	0	0.636	N/A
**Immunophenotypic features**							
BCL2	5	111	68.5 [55.2; 79.3]	5.0 [0.0; 80.2]	0	0.347	N/A
BCL6	5	112	92.0 [81.3; 96.8]	0.0 [0.0; 79.2]	0	0.139	N/A
c-Myc protein	4	85	76.8 [40.4; 94.2]	69.1 [10.6; 89.3]	0.611	0.0149	N/A
CD20	3	56	1.0 [0.0; 1.0]	0.0 [0.0; 89.6]	0	1	N/A
CD10	8	127	54.4 [38.9; 69.0]	19.0 [0.0; 61.7]	0.136	0.152	N/A
MUM1	7	95	70.5 [58.0; 80.6]	0.0 [0.0; 70.8]	0	0.295	N/A
Ki-67 ≥70%	6	53	83.1 [64.1; 93.1]	0.0 [0.0; 74.6]	0.0146	0.164	N/A
GCB	12	188	60.0 [46.3; 72.2]	0.0 [0.0; 58.3]	0.372	0.0013	0.219

**Table 7:** Diagnostic criteria, FISH methodology, and key IHC thresholds used in each study. This table summarizes the diagnostic criteria of “DHL” according to (1) the 2008 WHO Classification, (2) WHO-HAEM4, and (3) WHO-HAEM5. 2008 WHO Classification: B-cell lymphoma unclassifiable, intermediate between diffuse large B-cell lymphoma and Burkitt lymphoma (BCLU), with *MYC* and *BCL2* translocations [61]. WHO-HAEM4: all large B-cell lymphomas with *MYC* and *BCL2* and/or *BCL6* rearrangements except those fulfilling the diagnostic criteria for a follicular lymphoma or B-lymphoblastic leukemia/lymphoma (B-LBL) [62]. WHO-HAEM5: aggressive B-cell lymphomas with concurrent *MYC* and *BCL2* rearrangements, with or without *BCL6* rearrangement [7]. FP = fusion probe; BAP = break apart probe. N/A = Not available.

Study	Diagnostic Criteria	FISH Methodology	FISH Probe Used, Yes (Y)/No (N)					Threshold (%)			
			*IGH/MYC* FP	*IGH/BCL2* FP	*MYC* BAP	*BCL2* BAP	*BCL6* BAP	c-Myc	BCL2	BCL6	Ki-67
Wu et al., 2010 [33]	2008 WHO classification	N/A	N/A					N/A	N/A	N/A	N/A
Kobayashi et al., 2012 [34]	2008 WHO classification	*BCL6* break apart probe, *IGH/MYC* fusion probe, *IGH/BCL2* fusion probe	Y	Y	N	N	Y	N/A	N/A	N/A	N/A
Perry et al., 2013 [35]	2008 WHO classification	*MYC*, *BCL6*, *IGK*, *IGL* break apart probes, *IGH/MYC* fusion probe, *IGH/BCL2* fusion probe	Y	Y	Y	N	Y	N/A	N/A	N/A	N/A
Gebauer et al., 2013 [36]	2008 WHO classification	*MYC*, *BCL2*, *BCL6*, *IGH* break apart probes	N	N	Y	Y	Y	N/A	N/A	N/A	Values
Yoshida et al., 2015 [37]	2008 WHO classification	*MYC*, *BCL6* break apart probe, *IGH/BCL2* fusion probe	N	Y	Y	N	Y	N/A	30	30	Values
Ye et al., 2016 [38]	The applicable WHO classification between 1998 and 2010	*MYC*, *BCL2*, *BCL6*, *IGH* break apart probes, *IGH/MYC* fusion probe	Y	N	Y	Y	Y	N/A	N/A	N/A	70
Landsburg, Petrich et al., 2016 [39]	2008 WHO classification	N/A	N/A					N/A	N/A	N/A	90
Li et al., 2016 [40]	WHO-HAEM4	*MYC*, *BCL6* break apart probes, *IGH/BCL2* fusion probe	N	Y	Y	N	Y	40	50	30	70
Landsburg, Falkiewicz et al., 2016 [11]	2008 WHO classification	N/A	N/A					N/A	N/A	N/A	90
Roth et al., 2016 [41]	2008 WHO classification	N/A	N/A					N/A	N/A	N/A	N/A
Moench et al., 2016 [42]	2008 WHO classification	*MYC*, *BCL2*, *BCL6* break apart probes, *IGH/MYC* fusion probe, *IGH/BCL2* fusion probe	Y	Y	Y	Y	Y	N/A	N/A	N/A	Values
Oliveira et al., 2017 [43]	2008 WHO classification	*MYC*, *BCL2*, *BCL6* break apart probes, *IGH/MYC* fusion probe	Y	N	Y	Y	Y	50	70	50	90
Miyaoka et al., 2018 [44]	WHO-HAEM4	*MYC*, *BCL2*, *BCL6* break apart probes, *IGH/MYC* fusion probe	Y	N	Y	Y	Y	50	30	30	Values
Hancock et al., 2018 [45]	WHO-HAEM4	N/A	N/A					N/A	N/A	N/A	N/A
Ma et al. 2019 [46]	WHO-HAEM4	N/A	N/A					N/A	N/A	N/A	80
Zhang et al., 2020 [47]	WHO-HAEM4	*MYC*, *BCL6* break apart probes, *IGH/MYC* fusion probe, *IGH/BCL2* fusion probe	Y	Y	Y	N	Y	40	50	30	N/A
Ma et al., 2020 [48]	2008 WHO classification	*MYC*, *BCL2*, *BCL6* break apart probes	N	N	Y	Y	Y	N/A	N/A	N/A	70
Rodrigues-Fernandes et al., 2021 [49]	WHO-HAEM4	*MYC*, *BCL6* break apart probes, *IGH/BCL2* fusion probe	N	Y	Y	N	Y	40	50	30	Values
Tsai et al., 2021 [50]	WHO-HAEM4	*MYC*, *BCL2*, *BCL6* break apart probes	N	N	Y	Y	Y	40	50	30	70
Leskova et al., 2022 [51]	WHO-HAEM4	*MYC*, *BCL2*, *BCL6* break apart probes	N	N	Y	Y	Y	N/A	N/A	N/A	N/A
Almeida et al., 2022 [52]	The applicable WHO classification between 2010 and 2020	*MYC*, *BCL2*, *BCL6* break apart probes	N	N	Y	Y	Y	40	50	30	N/A
Alsuwaidan et al., 2022 [53]	WHO-HAEM4	*MYC*, *BCL6* break apart probes, *IGH/MYC* fusion probe, *IGH/BCL2* fusion probe	Y	Y	Y	N	Y	40	N/A	N/A	N/A
Liu et al., 2022 [54]	WHO-HAEM4	*MYC*, *BCL2*, *BCL6*, *IGH* break apart probes, *IGH/MYC* fusion probe, *IGH/BCL2* fusion probe	Y	Y	Y	Y	Y	40	50	30	N/A
Thirunavukkarasu et al., 2022 [55]	WHO-HAEM4	*MYC*, *BCL2*, *BCL6* break apart probes	N	N	Y	Y	Y	N/A	N/A	N/A	N/A
Blomme et al., 2024 [56]	WHO-HAEM5	*MYC*, *BCL2*, *BCL6* break apart probes, *MYC/BCL6* fusion probe	N	N	Y	Y	Y	N/A	N/A	N/A	N/A
Marianini et al., 2024 [57]	WHO-HAEM4	N/A	N/A					N/A	N/A	N/A	N/A
Taybi et al., 2024 [58]	The applicable WHO classification between 2013 and 2022	*MYC*, *BCL2*, *BCL6* break apart probes	N	N	Y	Y	Y	N/A	N/A	N/A	90
Sebastian et al., 2024 [59]	WHO-HAEM4	N/A	N/A					N/A	N/A	N/A	N/A
Kim et al., 2025 [60]	The applicable WHO classification between 2012 and 2021	*MYC*, *BCL6* break apart probes, *IGH/BCL2* fusion probe, *MYC/BCL6* fusion probe	N	Y	Y	N	Y	40	50	30	N/A

Three B-lymphoblastic leukemia/lymphoma (B-LBL) cases across two studies were subsequently found to harbor *MYC/BCL2* translocations [37,42]. B-LBL falls under the category of precursor lymphoid cells committed to the B-cell lineage, as opposed to DHL, which are mature B-cell neoplasms [7]. Two cases were excluded from the meta-analysis due to THL rearrangements [42], lack of testing for *BCL6* rearrangement for possible THL [37], and absence of IHC or flow cytometry for terminal deoxynucleotidyl transferase (TdT), CD34, or CD20 to confirm precursor or mature B-cell states [37]. The third case highlighted a rare situation, whereby immunoprofiling showed an immature phenotype [37], a recognized phenotype of DHL in WHO-HAEM5 [7].

Meta-analysis of morphological features between *MYC/BCL2* and *MYC/BCL6* DHL was not performed, as most morphological data (90%) was available for *MYC/BCL2* DHL cases only.

### 3.4. Cytogenetic and Molecular Features

FISH methodology for each study is summarized in Table 7. *MYC*, *BCL2,* and *BCL6* translocation partner genes were reported in 32, 12, and three cases, respectively, across five studies [34,35,42,44,54]. As expected, *IGH* was a more common translocation partner than non-*IGH* genes for *MYC* and *BCL2*, accounting for 30.0–85.7% and 85.7–100.0% of cases, respectively [34,35,42,44,54] (Supplementary Table S8). It should be noted that apart from *IGH/MYC* probes, break-apart, rather than dual-fusion probes, were used, which preclude the identification of non-*IGH* translocation partners, such as *IGL* and *IGK*. Likewise, the exclusive use of *BCL6* break-apart probes and the overall paucity of data limited the interpretation of *BCL6* translocation partners.

We could not perform a meta-analysis of EBV status, as it was only reported in a few studies and was often missing in the non-DHL group. EBV status was reported in seven studies using in situ hybridization (ISH) and IHC (Supplementary Table S9) [36,42,47,48,49,50]. One study was excluded from the meta-analysis as it included a mixed group of DHL and THL in the control group [54]. The remaining six studies included 96 patients [36,42,47,48,49,50]. Only one study detected any EBV-positive DHL, at a frequency of 6.7% (3/45 patients) [47].

### 3.5. Risk of Bias

Risk of bias assessment was performed using the National Institute of Health/National Heart, Lung, and Blood Institute (NIH/NHLBI) Quality Assessment Tool. The overall quality was fair in most included studies, except in two, which were considered poor [42,59] (Supplementary Tables S2 and S3). Eleven studies did not clearly define their case selection criteria, potentially creating selection bias. There was likely a degree of information or reporting bias because the pathologists assessing the immunophenotype were not blinded to translocation status in most studies. A small degree of measurement bias was likely in a small number of studies that did not report the threshold used to define positivity for IHC markers. Most studies did not report measures to adjust for confounding factors. Funnel plots and Egger’s test showed no significant publication bias in most meta-analyzed parameters (Table 4, Table 5 and Table 6, Supplementary Figure S1).

## 4. Discussion

This review aimed to collate and meta-analyze evidence from multiple studies to identify clinical and histopathological predictive features of DHL in HGBCL, which may inform patient stratification strategies for further FISH testing. Twenty-nine studies reporting routine diagnostic workup features for mature B-cell lymphomas and features proposed as a pre-screening strategy for FISH testing were included (Table 3).

### 4.1. Clinical Features

DHL patients were associated with indicators of more aggressive disease, compared to non-DHL patients, including IPI scores and LDH elevation, which is consistent with prior studies [3,7,13,63,64]. Elevated IPI scores in DHL patients are thought to partly be attributable to extranodal involvement, including CNS and BM involvement [7,65,66]. We found that DHL was not significantly associated with extranodal involvement compared to non-DHL, although most DHL patients (67.1%) had extranodal disease. Insufficient numbers of studies reporting CNS involvement in DHL and non-DHL groups limited meta-analysis. Across ten studies, we found CNS involvement in 14.8% of DHL cases, which is within the reported range of 9–50% in previous studies [3]. BM involvement was present in 38.8% of DHL patients. We found a non-significant positive association between BM involvement and DHL compared with non-DHL patients.

*MYC/BCL6* DHL patients were notably more likely to have extranodal disease than those with *MYC/BCL2* rearrangement. CNS involvement was also more likely in *MYC/BCL6* compared to *MYC/BCL2* DHL cases, which has not been reported previously. Some studies have shown an increased risk of CNS disease in non-GCB DLBCL [67,68]. It is possible that the increased risk of CNS involvement was related to the propensity of the non-GCB subtype in *MYC/BCL6* DHL. Further studies that adjust for COO, a potential confounding factor, are needed to evaluate the association between *MYC/BCL6* DHL and CNS involvement.

### 4.2. Histopathological Features

We found that the GCB subtype was significantly more likely in DHL compared to non-DHL patients (RR 1.21, *p* = 0.043), in keeping with previously published findings [69,70]. This could be related to *MYC/BCL2* being the predominant subtype of DHL, which we confirmed to be more likely of GCB subtype (RR 1.40, *p* = 0.016) compared to *MYC/BCL6* using the Hans algorithm [16].

Interestingly, by individually considering COO determinants according to the Hans algorithm between the DHL and non-DHL groups, and between the two DHL subtypes, we identified non-significant trends for CD10 positivity, MUM1/IRF4 negativity, and BCL6 positivity. This may reflect small sample sizes when meta-analyzing the individual COO determinants, reducing statistical power to detect significant differences.

We identified a near-significant trend for c-Myc protein positivity in DHL (RR 1.41, *p* = 0.051), which has previously been reported to predict *MYC* rearrangement with variable accuracies [71,72]. One reason why only a near-significant difference was observed may be due to the overlapping immunophenotypic profiles between DHL and other non-DHL HGBCLs. For instance, double-expressor lymphoma (DEL), which is more prevalent than DHL, is characterized by concurrent c-Myc and BCL2 IHC overexpression [2,73]. DEL inclusion in the non-DH HGBCL group may have attenuated the pooled effect size of c-Myc expression in DHL. There was no significant difference in c-Myc expression between the *MYC*/*BCL2* and *MYC*/*BCL6* DHL subtypes.

Previously published studies have highlighted the limited utility of using high Ki-67 in predicting DHL [2]. We similarly observed a positive, non-significant association between increased Ki-67 and DHL compared with non-DHL (RR 1.36, *p* = 0.71). Limited availability of Ki-67 data in non-DHL comparison groups restricted meta-analysis to three studies using a ≥90% Ki-67 positivity threshold. Prior studies using the ≥90% Ki-67 threshold have reported missing a significant proportion of DHL [2]. Further studies using a lower ≥70% threshold, which was commonly applied in the studies evaluating Ki-67 between *MYC/BCL2* and *MYC/BCL6* DHL, may improve sensitivity for finding DHL cases among HGBCLs.

The WHO-HAEM5 guidelines recommend a sequential strategy of assessing morphology first, followed by testing for *MYC* and *BCL2* rearrangements when diagnosing DHL, recognizing that *MYC/BCL2*-rearranged HGBCL may arise within several morphological patterns (including DLBCL, NOS, Burkitt lymphoma, and HGBCL, NOS with blastoid or Burkitt-like features) [7]. Although we observed that the so-called “DLBCL morphology” was most common (range 28.6–81.8%), followed by BCLU (range 0.0–66.7%), we could not determine whether DHL was significantly associated with any specific morphology. This was partly confounded by variation in definitions and terminology for HGBCL morphology over time.

Additionally, finding a clearly defined, non-DHL control group was challenging as we were limited to the non-DHL cases reported in the included studies. In these studies, non-DHL groups had been pre-selected based on morphology and, consequently, comparator groups comprised a variety of different non-DHL HGBCL with different morphologies and genetic aberrations. Morphology-controlled analyses between DHL and non-DHL cases were considered (for instance, by restricting analyses to “DLBCL, NOS” morphology only). However, as previously discussed, these morphological subtypes are poorly defined, demonstrate limited reproducibility, and, in the case of DLBCL, NOS, encompass heterogeneous entities, precluding morphology-controlled comparisons between DHL and non-DHL.

Future prospective studies using the standardized morphological definitions (from WHO-HAEM5) with systematic FISH DHL verification may help confirm these morphological associations. However, as written, even these morphological definitions are likely to be challenging to apply with any consistency.

It should be noted that a case initially classified as B-LBL based on morphology and immunoprofile was subsequently found to harbor a *MYC/BCL2* translocation [42]. WHO-HAEM5 recognizes that around 2% of DHL cases have reported immature immunophenotypes (TdT immunopositivity, weak/negative CD20, negative BCL6, and/or light chains), which was seen here [7,42]. Importantly, this is distinct from B-LBL, a *bona fide* precursor lymphoid cell neoplasm, in which genetic abnormalities, including *MYC/BCL2* rearrangements, constitute “desirable” exclusion criteria [7]. The overlapping features between DHL with an immature phenotype and B-LBL highlight a known diagnostic challenge and underscore the need for FISH testing to differentiate these two entities, where DHL is clinically suspected. Because cases of DHL with immature phenotype are rare and may require different treatment regimens compared to B-LBL [74], larger multi-center studies, beyond existing case reports, are needed to determine its clinical characteristics.

Our results corroborate previously identified features but do not identify any new features to add to an algorithm. Our analysis indicates there is considerable overlap between DHL and non-DHL for many parameters and shows that testing has, to date, varied between centers and even between individual cases. In addition, the observed associations were of modest effect size, meaning features we have identified should only ever be used to identify which cases should be screened by FISH, rather than to predict their translocation status independent of FISH. Thus, there is a need for integrated, standardized molecular testing, most likely comprising a universal HGBCL *MYC* FISH test, followed by *BCL2* FISH testing for MYC-rearranged cases to satisfy WHO-HAEM5 classification criteria, or *BCL2* and *BCL6* FISH testing for *MYC*-rearranged cases to satisfy ICC classification criteria [7,8]. The cost-effectiveness of such a universal FISH strategy will require analysis. While we considered a range of parameters, including currently assessed histopathological and immunophenotypic parameters, the implications of the application of artificial intelligence (AI) to digitally scanned histopathological images remain to be determined. Such an AI approach might identify predictive parameters, or combinations of parameters, which are not currently identified by pathologists. Ultimately, the identification of emerging targeted therapies, including additional-agent venetoclax and PROteolysis TArgeting Chimera (PROTAC) BCL6 degraders [75,76], will make identification of *MYC/BCL2* and *MYC/BCL6*-rearranged lymphomas essential.

### 4.3. Strengths and Limitations

Our study has several strengths. Through our literature search, we comprehensively incorporated evidence from multiple geographical regions and captured DHL cases in a more representative demographic context. We also applied stringent criteria to select only studies that performed *MYC*, *BCL2,* and *BCL6* FISH testing to confirm DHL diagnosis. No significant uniform publication bias across the evaluated markers was shown, as demonstrated by relative symmetry in funnel plots. Furthermore, to our knowledge, this is the first meta-analysis evaluating the clinical, histological, and immunophenotypic features of DHL, including the *MYC/BCL2* and *MYC/BCL6* DHL subtypes. There are relatively few studies reporting *MYC/BCL6* DHL due to its relatively low prevalence (8–20% of cases) and previous inclusion within the same category as *MYC/BCL2* DHL or THL [3,77].

These results, however, need to be interpreted cautiously. While GCB was identified as a predictive feature, the reproducibility of immunohistochemically assigned COO compared with gene expression profiling is, itself, limited, with 72–83% concordance [78,79]. This is due to a combination of innate lymphoma biology, the challenges of interobserver variability, and different pre-analytical processing of the sample [16,79,80]. Furthermore, protein expression patterns of c-Myc, BCL2, and BCL6 can identify an additional group of HGBCLs above and beyond those identified by FISH, indicating that rearrangement-driven and expression-driven aggressive HGBCL are only partly overlapping groups [69].

Results should be interpreted with consideration of inter-study heterogeneity. We used a random effects model, and used *I^2^* values (the percentages of total variation in effect estimates due to heterogeneity between studies rather than chance) to estimate statistical heterogeneity [29], which was low to moderate for several immunotypic marker comparisons (c-Myc, MUM1, GCB), but substantial to critical for other markers (BCL2, CD10, Ki-67). Similarly, tau^2^ values (estimating variance across all studies) were predominantly zero or low but higher for several markers (BCL2, CD10, Ki-67). Inter-study heterogeneity was likely driven by heterogeneity in the control groups used in different studies. As previously mentioned, we were limited by the non-DHL cases reported in the included studies, which included a variety of different non-DHL HGBCL, with different morphologies and genetic aberrations. These entities may have overlapping features with DHL, thus reducing the overall pooled effect size.

It should also be noted that all but one of the included studies were retrospective in nature, hence were subject to bias in selection and the presence of missing data. Our handling of missing data, using the available-case analysis approach, may also introduce bias in effect estimates if data were not missing at random [30].

For instance, we were not able to comment on *MYC* translocation partners, although the presence of *MYC* rearrangements involving non-*IGH* versus *IGH* translocation partners is now known to have different prognostic implications [81]. Seven out of the 21 studies with available FISH methodology data solely used *MYC*, *BCL2,* and *BCL6* break apart probes to identify *MYC*, *BCL2,* and *BCL6* gene rearrangement. The remaining studies utilized *IGH/MYC* and/or *IGH/BCL2* fusion probes, which will only identify translocation to *IGH*. Only five studies reported gene translocation partners (Supplementary Table S7). As routine interphase FISH is unable to detect some cryptic *MYC* or *BCL2* rearrangements, *MYC/BCL2* rearrangements will inevitably have been missed in a small subset of HGBLs [81]. In a small proportion of cases, complex karyotypes may also have proved difficult to interpret [64].

Results should also be interpreted with consideration of small-study effects due to the rarity of DHL cases. Small study sizes can result in wide confidence intervals around measured outcomes in individual studies and increase the uncertainty of the overall effect sizes when pooling different studies together. FISH has not historically been performed on all DLBCL or other HGBCL. Limited sample sizes also limited broader interpretation of results, including adjustment for potential clinical confounders such as patient age, sex, and geographic location. The WHO-HAEM5 guidelines, based on *MYC/BCL2* translocation, should facilitate the development of larger, more consistently defined cohorts for future studies.

Our meta-analysis also highlighted the lack of standardized IHC thresholds (Table 7). For the c-Myc protein, eight and two studies selected thresholds for labeling the case “positive” of 40% and 50% of cells, respectively. BCL2 protein expression cut-off values of 50%, 30%, and 70% of cells were selected in seven, two, and one studies, respectively, corroborating recognized variability in defining IHC positivity [2,82]. Establishing uniform inter-study thresholds, perhaps of 40% and 50% for c-Myc and BCL2, respectively, might improve reproducibility in IHC interpretation and enable more reliable comparisons between DHL and non-DHL, and between DHL subtypes. This is pragmatic because 40% and 50% thresholds for c-Myc and BCL2 are in common use for assessing so-called “double-expresser” and COO status [6,83].

Lastly, although this review identified clinical and pathological features associated with DHL, compared to non-DHL, amongst all HGBCLs, it is important to note that the purpose of identifying these associations is to help select a subset of patients for FISH testing to confirm the diagnosis of DHL, rather than attempting to make a diagnosis of DHL on the basis of predictive features without undertaking FISH.

## 5. Conclusions

This review correlates DHL with features of a more aggressive clinical disease course, including a higher IPI score and LDH. We found GCB immunophenotype as a histopathological feature with statistically significant predictive value for DHL. When comparing *MYC/BCL2* DHL predictors with *MYC/BCL6* DHL predictors, we additionally identified extranodal disease, CNS involvement, and non-GCB immunophenotype. However, none of these features could identify 100% of DHL, nor completely distinguish between *MYC/BCL2* and *MYC/BCL6-*rearranged cases.

Individual features that may warrant further investigation include c-Myc immunostaining, which just failed to reach statistical significance. Further studies that adjust for COO, a potential confounding factor, are needed to evaluate whether CNS involvement can predict *MYC/BCL6* DHL rather than *MYC/BCL2* DHL.

We also recommend standardizing IHC threshold values, perhaps utilizing existing widely adopted thresholds for c-Myc (40%), BCL2 (50%), and Ki-67 (≥70%). Such standardization will make the pooling of cohorts into larger studies more feasible, giving clearer answers to the questions that we tackled in this study.

## Figures and Tables

**Table 1:** Search strategy.

1	“double-hit” adj9 lymphoma* OR “HGBL”
2	B-cell lymphoma, unclassifiable, with features intermediate between DLBCL and Burkitt lymphoma
3	DHL AND lymphoma*
4	1 OR 2 OR 3
5	exp Lymphoma, Non-Hodgkin
6	(“non-Hodgkin” or “non Hodgkin”) AND (lymphoma*)
7	(“mature B-cell” or “mature b cell”) AND (lymphoma*)
8	(“diffuse large B-cell” or “diffuse large b cell”) AND (lymphoma*)
9	“DLBCL” AND lymphoma*
10	(“high grade” or “high-grade” or “aggressive”) AND (lymphoma*)
11	“HGBL” AND lymphoma*
12	5 OR 6 OR 7 OR 8 OR 9 OR 10 OR 11
13	4 AND 12
14	(“double hit” or “double-hit” or “concurrent”) AND (translocat* or rearrang*)
15	(“MYC” or “8q24.2”) AND (translocat* or rearrang*)
16	(“BCL” or “BCL2” or “BCL-2” or “18q21.3”) AND (translocat* or rearrang*)
17	(“BCL” or “BCL6” or “BCL-6” or “3q27”) AND (translocat* or rearrang*)
18	(MYC-IG or “MYC/IG” or MYC IG) AND (translocat* or rearrang*)
19	(t or translocat*) AND (14;18)
20	(IG or IGH) AND (translocation* or rearrangement*)
21	14 OR 15 OR 16 OR 17 OR 18 OR 19 OR 20
22	biological character*
23	morphol* or patholog* or histolog*
24	immunophenotype* or immunophenotypic analysis
25	clinical presentation or clinical feature* or symptom*
26	22 OR 23 OR 24 OR 25
27	(WHO classification) AND lymphoma*
28	(gene* or molecul* or cytogenetic*) AND (analy* or method*)
29	(immunohistochem* or IHC) or exp Immunohistochemistry/
30	florescence in situ hybridisation or exp In Situ Hybridization, Fluorescence/
31	immunostain*
32	CD10 or BCL2 or BCL-2 or BCL6 or BCL-6 or IRF4 or IRF-4 or MUM1
33	27 OR 28 OR 29 OR 30 OR 31 OR 32
34	21 OR 26 OR 33
35	13 AND 34

**Table 2:** Inclusion and exclusion criteria for study selection.

Domain	Inclusion Criteria	Exclusion Criteria
Population	Human subjects diagnosed with DHL in line with the 2016 update of WHO lymphoma classification, or studies prior to 2016 with clearly defined characteristics of DHL	Non-human subjects and in vitro studies, triple-hit lymphomas (THL) *, diffuse histiocytic lymphoma, low-grade or non-mature B-cell lymphoid proliferations, studies in which FISH was not performed on all of *MYC*, *BCL2* and *BCL6*
Outcomes	Clinicopathological, morphological and cytogenetic features of DHL	Wrong outcomes include protocols for relapse of disease, novel treatment regimens or stem cell transplant alone, molecular findings or classification only
Comparison	Aggressive or high-grade B-cell lymphoid proliferations and lymphomas that do not harbor concurrent *MYC* and *BCL2* and/or *BCL6* translocations	Low-grade B-cell or non-mature B-cell lymphoid proliferations
Study type	Any original studies with *n* ≥ 3	Small sample size (*n* < 3), reviews, conference abstracts, comments and editorials

* Triple-hit lymphomas (THL) are defined as high-grade B-cell lymphomas with concomitant *MYC*, *BCL2* and *BCL6* rearrangements.

**Table 3:** Study design and patient demographics of included studies. N/A = Not applicable.

Study	Location	Study Design	No. of DHL Patients			Median Age of DHL Patients	Male DHL Patients		No. of Non-DHL Patients	Non-DHL Group
			Total	*MYC/BCL2*	*MYC/BCL6*		No.	%		
Wu et al., 2010 [33]	USA	Case series	10	9	1	52.5	7	70	0	N/A
Kobayashi et al., 2012 [34]	Japan	Retrospe-ctive cohort	3	1	2	66	N/A	N/A	28	Non-DH DLBCL
Perry et al., 2013 [35]	USA	Case series	11	N/A	N/A	N/A	N/A	N/A	0	N/A
Gebauer et al., 2013 [36]	Germany	Retrospe-ctive cohort	29	13	16	69 (*MYC/BCL2*: 62; *MYC/BCL6*: 71.5)	19	65.5	16	Non-DH Burkitt lymphoma
Yoshida et al., 2015 [37]	Japan	Case series	14	13	1	65	11	78.6	0	N/A
Ye et al., 2016 [38]	USA	Case series	33	20	13	N/A	20	60.6	0	N/A
Landsburg, Petrich et al., 2016 [39]	USA	Case series	92	76	16	N/A	N/A	N/A	0	N/A
Li et al., 2016 [40]	USA	Case series	157	157	0	61	103	65.6	0	N/A
Landsburg, Falkiewicz et al., 2016 [11]	USA	Retrospe-ctive cohort	45	N/A	N/A	60	24	53.3	236	Non-DH DLBCL/BCLU
Roth et al., 2016 [41]	USA	Retrospe-ctive cohort	16	11	5	*MYC/BCL2*: 64; *MYC/BCL6*: 69	N/A	N/A	0	N/A
Moench et al., 2016 [42]	USA	Case series	9	8	1	61	5	55.6	0	N/A
Oliveira et al., 2017 [43]	Brazil	Retrospe-ctive cohort	3	2	1	65	1	33.30%	87	Non-DH BL and DLBCL
Miyaoka et al., 2018 [44]	Japan	Retrospe-ctive cohort	11	11	0	66	6	54.5	0	N/A
Hancock et al., 2018 [45]	USA	Case series	13	7	6	73 (*MYC/BCL2*: 81; *MYC/BCL6*: 72)	7	53.8	0	N/A
Ma et al., 2019 [46]	China	Retrospe-ctive cohort	26	14	12	N/A	13	50	101	DLBCL (Double copy number gain)
Zhang et al., 2020 [47]	China	Case series	48	14	34	51.5 (*MYC/BCL2*: 51.5; *MYC/BCL6*: 52.5)	29	60.4	0	N/A
Ma et al., 2020 [48]	China	Case series	14	6	8	N/A	11	78.6	0	N/A
Rodrigues-Fernandes et al., 2021 [49]	Brazil	Case series	5	3	2	72 (*MYC/BCL2*: 67; *MYC/BCL6*: 72.5)	2	40	0	N/A
Tsai et al., 2021 [50]	Taiwan	Retrospe-ctive cohort	20	8	12	N/A	14	70	258	Non-DH DLBCL
Leskova et al., 2022 [51]	Slovakia	Case series	56	40	16	N/A	N/A	N/A	0	N/A
Almeida et al., 2022 [52]	Portugal	Retrospe-ctive cohort	16	14	2	*MYC/BCL2*: 68; *MYC/BCL6*: 58	7	43.8	24	HGBL
Alsuwaidan et al., 2022 [53]	USA	Retrospe-ctive cohort	34	21	13	*MYC/BCL2*: 63; *MYC/BCL6*: 56	21	61.8	39	DLBCL (absence of *MYC* rearrangeme-nts by FISH)
Liu et al., 2022 [54]	USA, China	Case series	14	8	6	65 (*MYC/BCL2*: 65.5; *MYC/BCL6*: 51.5)	6	42.9	0	N/A
Thirunavukkarasu et al., 2022 [55]	India	Retrospe-ctive cohort	3	3	0	72.3	2	66.7	8	Non-DH HGBL
Blomme et al., 2024 [56]	Belgium	Retrospe-ctive cohort	11	0	11	68	5	45.5	115	Non-DH DLBCL/HGBL
Marianini et al., 2024 [57]	France	Retrospe-ctive cohort	7	4	3	76 (*MYC/BCL2*: 78; *MYC/BCL6*: 66)	1	14.3	1	Non-DH DLBCL
Taybi et al., 2024 [58]	Morocco	Retrospe-ctive cohort	3	0	3	48	3	100.0	46	DLBCL/HGBL, NOS
Sebastian et al., 2024 [59]	India	Prospect-ive cohort	10	8	2	N/A	N/A	N/A	61	Non-DH HGBL
Kim et al., 2025 [60]	USA	Retrospe-ctive cohort	60	0	60	66	33	55.0	217	DLBCL, NOS

## Data Availability

The original contributions presented in this study are included in the article/Supplementary Materials. Further inquiries can be directed to the corresponding author.

## Supplementary Materials

The following supporting information can be downloaded at: https://www.mdpi.com/article/10.3390/biomedicines14061375/s1, Table S1: Data Extraction Form Template; Figure S1: Funnel plots; Table S1: Search strategy; Table S2: Inclusion and exclusion criteria for study selection; Table S3: Risk of bias using the National Institute of Health/National Heart, Lung, and Blood Institute (NIH/NHLBI) Quality Assessment Tool; Table S4: Proportions of non-meta-analysed IHC and flow cytometry markers; Table S5: Morphological classification of DHL cases; Table S6: Translocation partner genes for *MYC*, *BCL2* and *BCL6*; Table S7: EBV positivity in DHL patients; Table S8: PRISMA 2020 checklist.
