# Supplementary material for: Clinical and Pathological Features That Predict High-Grade B-Cell Lymphomas (HGBCLs) with MYC and BCL2 or BCL6 Translocations (Double-Hit Lymphoma): A Systematic Review and Meta-Analysis
Source: Biomedicines. 2026 Jun 18;14(6):1375. doi: 10.3390/biomedicines14061375 (PMC13297255; doi:10.3390/biomedicines14061375)

Table S1: PRISMA 2020 Checklist.

Section and Topic	Item #	Checklist item	Location where item is reported
**TITLE**			
Title	1	Identify the report as a systematic review.	Title
**ABSTRACT**			
Abstract	2	See the PRISMA 2020 for Abstracts checklist.	Abstract
**INTRODUCTION**			
Rationale	3	Describe the rationale for the review in the context of existing knowledge.	Introduction
Objectives	4	Provide an explicit statement of the objective(s) or question(s) the review addresses.	Introduction (last paragraph)
**METHODS**			
Eligibility criteria	5	Specify the inclusion and exclusion criteria for the review and how studies were grouped for the syntheses.	Materials and Methods (Table 2)
Information sources	6	Specify all databases, registers, websites, organisations, reference lists and other sources searched or consulted to identify studies. Specify the date when each source was last searched or consulted.	Materials and Methods (2.1. Search Strategy)
Search strategy	7	Present the full search strategies for all databases, registers and websites, including any filters and limits used.	Materials and Methods (2.1. Search Strategy, Table 1)
Selection process	8	Specify the methods used to decide whether a study met the inclusion criteria of the review, including how many reviewers screened each record and each report retrieved, whether they worked independently, and if applicable, details of automation tools used in the process.	Materials and Methods (2.1. Search Strategy)
Data collection process	9	Specify the methods used to collect data from reports, including how many reviewers collected data from each report, whether they worked independently, any processes for obtaining or confirming data from study investigators, and if applicable, details of automation tools used in the process.	Materials and Methods (2.1. Search Strategy)
Data items	10a	List and define all outcomes for which data were sought. Specify whether all results that were compatible with each outcome domain in each study were sought (e.g. for all measures, time points, analyses), and if not, the methods used to decide which results to collect.	Materials and Methods (2.3. Data Extraction); Supplementary Table S2
	10b	List and define all other variables for which data were sought (e.g. participant and intervention characteristics, funding sources). Describe any assumptions made about any missing or unclear information.	Materials and Methods (2.3. Data Extraction); Supplementary Table S2
Study risk of bias assessment	11	Specify the methods used to assess risk of bias in the included studies, including details of the tool(s) used, how many reviewers assessed each study and whether they worked independently, and if applicable, details of automation tools used in the process.	Materials and Methods (2.5. Risk of Bias Assessment)

Section and Topic	Item #	Checklist item	Location where item is reported
Effect measures	12	Specify for each outcome the effect measure(s) (e.g. risk ratio, mean difference) used in the synthesis or presentation of results.	Materials and Methods (2.4. Statistical Analyses)
Synthesis methods	13a	Describe the processes used to decide which studies were eligible for each synthesis (e.g. tabulating the study intervention characteristics and comparing against the planned groups for each synthesis (item #5)).	Results (3.1. Study Selection and Characteristics)
	13b	Describe any methods required to prepare the data for presentation or synthesis, such as handling of missing summary statistics, or data conversions.	Materials and Methods (2.4. Statistical Analyses); Results
	13c	Describe any methods used to tabulate or visually display results of individual studies and syntheses.	Materials and Methods (2.3. Data Extract, 2.4. Statistical Analyses)
	13d	Describe any methods used to synthesize results and provide a rationale for the choice(s). If meta-analysis was performed, describe the model(s), method(s) to identify the presence and extent of statistical heterogeneity, and software package(s) used.	Materials and Methods (2.4. Statistical Analyses)
	13e	Describe any methods used to explore possible causes of heterogeneity among study results (e.g. subgroup analysis, meta-regression).	Materials and Methods (2.4. Statistical Analyses)
	13f	Describe any sensitivity analyses conducted to assess robustness of the synthesized results.	Materials and Methods (2.4. Statistical Analyses)
Reporting bias assessment	14	Describe any methods used to assess risk of bias due to missing results in a synthesis (arising from reporting biases).	Materials and Methods (2.5. Risk of Bias Assessment)
Certainty assessment	15	Describe any methods used to assess certainty (or confidence) in the body of evidence for an outcome.	Materials and Methods (2.4. Statistical Analyses)
**RESULTS**			
Study selection	16a	Describe the results of the search and selection process, from the number of records identified in the search to the number of studies included in the review, ideally using a flow diagram.	Results (Figure 1)
	16b	Cite studies that might appear to meet the inclusion criteria, but which were excluded, and explain why they were excluded.	Results (3.2. Clinical

Section and Topic	Item #	Checklist item	Location where item is reported
			Features, 3.3. Histopathological Features, 3.4. Cytogenetic and Molecular Features)
Study characteristics	17	Cite each included study and present its characteristics.	Results (Table 3)
Risk of bias in studies	18	Present assessments of risk of bias for each included study.	Supplementary Tables S3-4
Results of individual studies	19	For all outcomes, present, for each study: (a) summary statistics for each group (where appropriate) and (b) an effect estimate and its precision (e.g. confidence/credible interval), ideally using structured tables or plots.	Results (Figures 2-3, Tables 4-7); Supplementary Materials (Tables S5-9)
Results of syntheses	20a	For each synthesis, briefly summarise the characteristics and risk of bias among contributing studies.	Results
	20b	Present results of all statistical syntheses conducted. If meta-analysis was done, present for each the summary estimate and its precision (e.g. confidence/credible interval) and measures of statistical heterogeneity. If comparing groups, describe the direction of the effect.	Results (Figures 2-3, Tables 4-7)
	20c	Present results of all investigations of possible causes of heterogeneity among study results.	Results (Figures 2-3, Tables 4-7)
	20d	Present results of all sensitivity analyses conducted to assess the robustness of the synthesized results.	Results (Figures 2-3, Tables 4-7)
Reporting biases	21	Present assessments of risk of bias due to missing results (arising from reporting biases) for each synthesis assessed.	Supplementary Materials (Tables S3-4)
Certainty of evidence	22	Present assessments of certainty (or confidence) in the body of evidence for each outcome assessed.	Results (Figures 2-3, Tables 4-7)
**DISCUSSION**			
Discussion	23a	Provide a general interpretation of the results in the context of other evidence.	Discussion (4.1. Clinical Features, 4.2. Histopathological Features)
	23b	Discuss any limitations of the evidence included in the review.	Discussion (4.3. Strength and Limitations)
	23c	Discuss any limitations of the review processes used.	Discussion (4.3. Strength and Limitations)
	23d	Discuss implications of the results for practice, policy, and future research.	Discussion and Conclusions

Section and Topic	Item #	Checklist item	Location where item is reported
**OTHER INFORMATION**			
Registration and protocol	24a	Provide registration information for the review, including register name and registration number, or state that the review was not registered.	Materials and Methods (2.1. Search Strategies)
	24b	Indicate where the review protocol can be accessed, or state that a protocol was not prepared.	Materials and Methods (2.1. Search Strategies)
	24c	Describe and explain any amendments to information provided at registration or in the protocol.	Materials and Methods (2.1. Search Strategies)
Support	25	Describe sources of financial or non-financial support for the review, and the role of the funders or sponsors in the review.	Funding
Competing interests	26	Declare any competing interests of review authors.	Conflicts of Interest
Availability of data, code and other materials	27	Report which of the following are publicly available and where they can be found: template data collection forms; data extracted from included studies; data used for all analyses; analytic code; any other materials used in the review.	Data Availability Statement

From: Page MJ, McKenzie JE, Bossuyt PM, Boutron I, Hoffmann TC, Mulrow CD, et al. The PRISMA 2020 statement: an updated guideline for reporting systematic reviews. BMJ 2021;372:n71. doi: 10.1136/bmj.n71.
This work is licensed under CC BY 4.0. To view a copy of this license, visit https://creativecommons.org/licenses/by/4.0/

Table S2: Data Extraction Form Template

**Samples**				
non_DHL_group	DHL_group_n	non_DHL_group_n	bcl2_n	bcl6_n
DHL_group_ denovo	DHL_transformed	DHL_previous		
**Ann Arbor stage**				
DHL_group_Ann_Arbor_Stage1 or2	DHL_group_Ann_ Arbor_Stage3or4	non_DHL_group_ Ann_Arbor_Stage1or2	non_DHL_group_Ann_Arbor_ Stage3or4	
**IPI score**				
DHL_group_IPI_0_2	DHL_group_IPI_3_5	non_DHL_group_IPI_0_2	non_DHL_group_IPI_3_5	
**LDH level**				
DHL_group_LDH_Elevated	DHL_group_LDH_normal	non_DHL_group_LDH_Elevated	non_DHL_group_LDH_normal	
**Extranodal involvement**				
DHL_group_extranodal_disease _ present	DHL_group_ extranodal_disease_absent	DHL_group_ extranodal_disease_over2sites	DHL_group_ extranodal_disease_under2sites	non_DHL_group_extranodal_ disease_present
non_DHL_group_extranodal_ disease_absent	non_DHL_group_ extranodal_disease_over2sites	non_DHL_group_ extranodal_disease_under2sites		
**CNS & BM involvement**				
DHL_group_CNS_present	DHL_group_CNS_absent	non_DHL_group_CNS_present	non_DHL_group_CNS_absent	DHL_group_BM_present
DHL_group_BM_absent	non_DHL_group_BM_present	non_DHL_group_BM_absent		
**B symptom**				
DHL_group_ Bsymptoms_present	DHL_group_ Bsymptoms_absent	non_DHL_group_ Bsymptoms_present	non_DHL_group_Bsymptoms_ absent	
**ECOG PS**				

DHL_group_ECOG_PS_0_1	DHL_group_ECOG_PS_2_4	non_DHL_group_ECOG_PS_0_1	non_DHL_group_ECOG_PS_2_4	DHL_group _ECOG_PS_0_2
DHL_group _ECOG_PS_3_5	non_DHL_group _ECOG_PS_0_2	non_DHL_group _ECOG_PS_3_5		
**CD19 expression (IHC)**				
CD19_cut_off	DHL_group_CD19_IHC_positive	DHL_group_CD19_IHC_negative	non_DHL_group_CD19_IHC_positive	non_DHL_group_CD19_IHC_negative
**CD19 expression (flow cytometry)**				
DHL_group_CD19_positive	DHL_group_CD19_dim/decreased	DHL_group_CD19_variable	DHL_group_CD19_actual_negative	DHL_group_CD19_negative
non_DHL_group_CD19_positive	non_DHL_group_CD19_negative	non_DHL_group_CD19_dim	non_DHL_group_CD19_variable	
**CD20 expression (IHC)**				
CD20 cut_off	DHL_group_CD20_IHC_positive	DHL_group_CD20_IHC_negative	non_DHL_group_CD20_IHC_positive	non_DHL_group_CD20_IHC_negative
**CD20 expression (flow cytometry)**				
DHL_group_CD20_positive	DHL_group_CD20_dim/decreased	DHL_group_CD20_variable	DHL_group_CD20_negative	non_DHL_group_CD20_positive
non_DHL_group_CD20_dim	non_DHL_group_CD20_variable			
**CD10 expression (flow cytometry)**				
DHL_group_CD10_positive	DHL_group_CD10_dim/decreased	DHL_group_CD10_negative	non_DHL_group_CD10_positive	non_DHL_group_CD10_negative
**CD45 expression (flow cytometry)**				
DHL_group_CD45_positive	DHL_group_CD45_dim/decreased	DHL_group_CD45_acutal_negative	DHL_group_CD45_negative	non_DHL_group_CD45_positive

non_DHL_group_CD45_dim	non_DHL_group_CD45_negativ e			
**Light chain expression (flow cytometry)**				
DHL_group_light_chains_ monotypic_λ	DHL_group_light_ chains_normal_λ	DHL_group_light_ chains_λ_ Dim/decreased/ absent	DHL_group_light_chains_ monotypic_κ	DHL_group_light_chains_normal_κ
DHL_group_light_chains_κ_abs ent/negative	DHL_group_light_chains_κ_ Dim/decreased/absent			
**TdT expression (flow cytometry)**				
DHL_group_TdT_positive	DHL_group_TdT_negative	non_DHL_group_ TdT_positive/negative		
**CD34 expression (flow cytometry)**				
DHL_group_CD34_positive	DHL_group_CD34_negative	non_DHL_group_CD34_ positive/negative		
**MYC expression**				
MYC_cut_off	DHL_group_MYC_positive	DHL_group_MYC_negative	non_DHL_group_MYC_positive	non_DHL_group_MYC_negative
**BCL2 expression**				
BCL2_cut _off	DHL_group_BCL2_positive	DHL_group_BCL2_negative	non_DHL_group_BCL2_positive	non_DHL_group_BCL2_negative
**BCL6 expression**				
BCL6_cut_off	DHL_group_BCL6_positive	DHL_group_BCL6_negative	non_DHL_group_BCL6_positive	non_DHL_group_BCL6_negative
**Ki67 expression**				
Ki67 _cut_off	DHL_group_Ki67_positive	DHL_group_Ki67_negative	DHL_group_Ki67_positive_70	DHL_group_Ki67_negative_70
DHL_group_Ki67_positive_90	DHL_group_Ki67_ negative_90	non_DHL_group_ Ki67_positive	non_DHL_group_Ki67_negative	non_DHL_group_Ki67_positive_70

non_DHL_group_Ki67_negative_70	non_DHL_group_Ki67_positive_90	non_DHL_group_Ki67_negative_90		
**CD10 expression**				
CD10 _cut_off	DHL_group_CD10_IHC_positive	DHL_group_CD10_IHC_negative	non_DHL_group_CD10_IHC_positive	non_DHL_group_CD10_IHC_negative
**MUM1/IRF4 expression**				
MUM1_cut_off	DHL_group_MUM1_positive	DHL_group_MUM1_negative	non_DHL_group_MUM1_positive	non_DHL_group_MUM1_negative
**Pax5 expression**				
Pax5_cut_off	DHL_group_Pax5_positive	DHL_group_Pax5_negative	non-DHL_group_Pax5_positive	non-DHL_group_Pax5_negative
**CD79a expression**				
CD79a_cut_off	DHL_group_CD79a_positive	DHL_group_CD79a_negative	non_DHL_group_CD79a_positive	non_DHL_group_CD79a_negative
**TdT expression**				
TdT_cut_off	DHL_group_TdT_positive	DHL_group_TdT_negative	non_DHL_group_TdT_positive	non_DHL_group_TdT_negative
**TP53 expression**				
TP53_cut_off	DHL_group_TP53_positive	DHL_group_TP53_negative	non_DHL_group_TP53_positive	non_DHL_group_TP53_negative
**TP53 gene mutation status**				
Detection method	DHL_group_TP53_abnormal	DHL_group_TP53_normal	non_DHL_group_TP53_abnormal	non_DHL_group_TP53_normal
**Translocation partner**				
MYC_translocation_partner_nonIGH	MYC_translocation_partner_IGH14q32	MYC_translocation_partner_IGL22q11	MYC_translocation_partner_IGK	BCL2_translocation_partner_IGH
BCL2_translocation_partner_others	BCL6_translocation_partner_IGH	BCL6_translocation_partner_others		

**Presenting sites**				
DHL_group_presenting_site_oropharynx	DHL_group_presenting_site_oral	DHL_group_presenting_site_nasopharynx	DHL_group_presenting_site_pharyngeal	DHL_group_presenting_site_neck
DHL_group_presenting_site_stomach	DHL_group_presenting_site_soft_tissue	DHL_group_presenting_site_skeletal_muscle	DHL_group_presenting_site_brain	DHL_group_presenting_site_lymph_node
DHL_group_presenting_site_pelvis	DHL_group_presenting_site_small_intestine	DHL_group_presenting_site_bone	DHL_group_presenting_site_large_intestine	DHL_group_presenting_site_retroperitoneal
DHL_group_presenting_site_abdominal	DHL_group_presenting_site_breast	DHL_group_presenting_site_testicle	DHL_group_presenting_site_adrenal	DHL_group_presenting_site_bone_marrow/peripheral_blood
DHL_group_presenting_site_groin	DHL_group_presenting_site_skin	DHL_group_presenting_site_kidney	DHL_group_presenting_site_bladder	DHL_group_presenting_site_pancreas
DHL_group_presenting_site_liver	DHL_group_presenting_site_lung	DHL_group_presenting_site_pleural_fluid	DHL_group_presenting_site_thyroid	non_DHL_group_presenting_site_nasopharynx
non_DHL_group_presenting_site_soft_tissue	non_DHL_group_presenting_site_lymph_node	non_DHL_group_presenting_site_skeletal_muscle	non_DHL_group_presenting_site_peritoneal	non_DHL_group_presenting_site_small_intestine
non_DHL_group_presenting_site_large_intestine	non_DHL_group_presenting_site_liver	non_DHL_group_presenting_site_pleural_fluid	non_DHL_group_presenting_site_thyroid	
**Morphology**				
DHL_group_morphology_GCB	DHL_group_morphology_Non_GCB	DHL_group_morphology_DLBCL	DHL_group_morphology_BL	DHL_group_morphology_burkitt_like
DHL_group_morphology_BCLU	DHL_group_morphology_B-LBL	DHL_group_morphology_HGBL	DHL_group_morphology_DLBCL+FL	DHL_group_morphology_CD10+ B-cell neoplasm, DLBCL or Burkitt lymphoma, or lymphoblastic leukemia

non_DHL_group_morphology_GCB	non_DHL_group_morphology_Non_GCB	non_DHL_group_morphology_DLBCL	non_DHL_group_morphology_BL	non_DHL_group_morphology_burkitt_like
non_DHL_group_morphology_BCLU	non_DHL_group_morphology_B-LBL	non_DHL_group_morphology_HGBL	non_DHL_group_morphology_FL	non_DHL_group_morphology_others

Table S3: Risk of bias using the National Institute of Health/National Heart, Lung, and Blood Institute (NIH/NHLBI) Quality Assessment Tool for Case Series Studies.

	1. Clearly stated objective	2. Clearly defined population	3. Consecutive cases	4. Comparable subjects	5. Clearly described intervention described	6. Valid outcomes	7. Adequate follow-up	8. Well-described statistical methods	9. Well-described results	Overall quality
Wu et al., 2010 [33]	Y	Y	Y	Y	NA	NA	NA	NR	Y	Fair
Perry et al., 2013 [35]	Y	Y	Y	CD	Y	Y	Y	Y	Y	Good
Yoshida et al., 2015 [37]	Y	Y	N	Y	Y	Y	N	Y	Y	Fair
Ye et al., 2016 [38]	Y	Y	Y	CD	Y	Y	Y	Y	Y	Good
Landsburg, Petrich et al., 2016 [39]	Y	Y	Y	CD	N	Y	Y	Y	Y	Fair
Li et al., 2016 [40]	Y	Y	Y	CD	Y	Y	Y	Y	Y	Good
Roth et al., 2016 [41]	Y	Y	N	CD	Y	Y	Y	Y	Y	Fair
Moench et al., 2016 [42]	Y	Y	N	Y	Y	CD	N	NR	Y	Poor
Hancock et al., 2018 [45]	Y	Y	Y	Y	Y	Y	Y	NR	Y	Good
Zhang et al., 2020 [47]	Y	Y	Y	Y	Y	Y	N	Y	Y	Fair
Ma et al., 2020 [48]	Y	Y	Y	CD	N	Y	Y	Y	Y	Fair
Rodrigues-Fernandes et al., 2021 [49]	Y	Y	Y	Y	N	Y	Y	Y	Y	Fair
Leskova et al., 2022 [51]	Y	Y	Y	CD	NA	NA	NA	NR	Y	Fair
Liu et al., 2022 [54]	Y	Y	N	Y	Y	Y	Y	Y	Y	Good

Table S4. Risk of bias using the National Institute of Health/National Heart, Lung, and Blood Institute (NIH/NHLBI) Quality Assessment Tool for Observational Cohort and Cross-Sectional Studies.

	1. Clearly stated research objective	2. Clearly defined study population	3. Participation rate ≥ 50%	4. Recruitment from the similar population	5. Sample size justification	6. Sufficient timeframe to see effect	7. Clearly defined outcome measures	8. Blinding of outcome assessors	9. Loss to follow-up ≤ 20%	10. Confounding variables measured and adjusted	Overall quality
Kobayashi et al., 2012 [34]	Y	Y	Y	Y	N	NA	Y	NR	NA	NR	Fair
Gebauer et al., 2013 [36]	Y	Y	N	Y	N	NA	N	Y	NA	NR	Fair
Landsburg, Falkiewicz et al., 2016 [11]	Y	Y	Y	Y	N	NA	Y	NR	NA	Y	Fair
Oliveira et al., 2017 [43]	Y	Y	N	Y	N	NA	Y	NR	N	NR	Fair
Miyaoka et al., 2018 [44]	Y	Y	N	Y	N	Y	Y	NR	N	NR	Fair

Ma et al., 2019 [46]	Y	Y	N	Y	N	NA	Y	NR	NA	NR	Fair
Tsai et al., 2021 [50]	Y	Y	Y	Y	N	NA	Y	NR	NA	NR	Fair
Almeida et al., 2022 [52]	Y	Y	Y	Y	N	NA	Y	NR	NA	NR	Fair
Alsuwaidan et al., 2022 [53]	Y	Y	Y	Y	N	NA	CD	NR	NA	NR	Fair
Thirunavukkarasu et al., 2022 [55]	Y	Y	Y	Y	N	NA	Y	NR	NA	NR	Fair
Blomme et al., 2024 [56]	Y	Y	Y	Y	N	NA	Y	NR	NA	NR	Fair
Marianini et al., 2024 [57]	Y	Y	N	Y	N	Y	Y	NR	Y	NR	Fair
Taybi et al., 2024 [58]	Y	Y	N	Y	N	Y	Y	NR	N	NR	Fair
Sebastian et al., 2024 [59]	Y	Y	N	Y	N	NA	N	NR	NA	NR	Poor
Kim et al., 2025 [60]	Y	Y	Y	Y	N	NA	Y	NR	NA	NR	Fair

Table S5: Proportions of non-meta-analysed IHC markers.

Study	CD19		PAX5		CD79a		TdT		p53	
	+	-	+	-	+	-	+	-	+	-
Moench et al., 2016 [42]	N/A	N/A	3	0	1	0	2	7	N/A	N/A
Ma et al., 2020 [48]	N/A	N/A	N/A	N/A	N/A	N/A	N/A	N/A	12	2
Zhang et al., 2020 [47]	29	2	N/A	N/A	N/A	N/A	N/A	N/A	N/A	N/A
Liu et al., 2022 [54]	8	0	7	1	6	0	0	6	7	3
Total	37	2	9	1	6	0	1	13	19	5

Table S6. Proportions of non-meta-analysed flow cytometry markers.

Study	CD19			CD20					CD10			CD45			Light chain				TdT		CD34	
	+	↓	Variable	-	+	↓	Variable	-	+	↓	-	+	↓	-	Monotypic λ	↓/-	Monotypic κ	↓/-	+	-	+	-
Wu et al., 2010 [33]	7	3	0	N/A	1	9	0	0	9	1	0	7	3	0	1	4	0	5	0	4	0	1
Roth et al., 2016 [41]	0	6	6	0	0	6	4	0	13	0	2	0	6	6	0	0	0	7	N/A	N/A	N/A	N/A
Moench et al., 2016 [42]	9	0	0	0	8	0	0	1	9	0	0	9	0	0	3	0	5	1	1	2	0	2
Liu et al., 2022 [54]	8	0	0	0	13	0	0	1	11	0	3	2	0	1	4	2	2	4	N/A	N/A	N/A	N/A

"+": Positive

"-": Negative

"↓": Dim/decreased

Table S7: Morphological classification of DHL cases.

Study	Morphology																	
	**DLBCL** *(n/%)*		**Burkitt** *(n/%)*		**Burkitt-like** *(n/%)*		**BCLU** *(n/%)*		**B-LBL** *(n/%)*		**DLBCL+FL** *(n/%)*		**HGBL** *(n/%)*		**Others** *(n/%)*		**Total** *(n/%)*	
Wu et al., 2010 [34]	3	30.0	2	20.0	0	0.0	4	40.0	0	0.0	0	0.0	0	0.0	1	10.0	10	100
Kobayashi et al., 2012 [34]	1	33.3	0	0.0	2	66.7	0	0.0	0	0.0	0	0.0	0	0.0	0	0.0	3	100
Gebauer et al., 2013 [36]	9	31.0	0	0.0	0	0.0	20	69.0	0	0.0	0	0.0	0	0.0	0	0.0	29	100
Yoshida et al., 2015 [37]	4	28.6	1	7.1	0	0.0	9	64.3	0	0	0	0.0	0	0.0	0	0.0	14	100
Li et al., 2016 [40]	92	58.6	0	0.0	0	0.0	61	38.9	0	0.0	4	2.5	0	0.0	0	0.0	157	100
Moench et al., 2016 [42]	3	33.3	0	0.0	0	0.0	3	33.3	1	11.1	0	0.0	2	22.2	0	0.0	9	100
Oliveira et al., 2017 [43]	2	66.7	1	33.3	0	0.0	0	0.0	0	0.0	0	0.0	0	0.0	0	0.0	3	100
Miyaoka et al., 2018 [44]	9	81.8	0	0.0	2	18.2	0	0.0	0	0.0	0	0.0	0	0.0	0	0.0	11	100
Almeida et al., 2022 [52]	9	56.3	0	0.0	0	0.0	6	37.5	0	0.0	0	0.0	0	0.0	1	6.3	16	100

Table S8: Translocation partner genes for *MYC*, *BCL2* and *BCL6*.

	MYC			***BCL2***			***BCL6***			**FISH probes**
	IGH (%)	Non-*IGH* (%)	Total	*IGH* (%)	Non-*IGH* (%)	Total	IGH (%)	Non-*IGH* (%)	Total	
Kobayashi et al., 2012 [34]	2 (66.7%)	1 (33.3%)	3	1 (100.0%)	0 (0.0%)	1	1 (50.0%)	1 (50.0%)	2	*IGH/MYC* fusion probe, *IGH/BCL2* fusion probe, *BCL6* break apart probe
Perry et al., 2013 [35]	7 (77.8%)	2 (22.2%)	9	N/A	N/A	N/A	N/A	N/A	N/A	*MYC*, *BCL6*, *IGK*, *IGL* break apart probes, *IGH/MYC* fusion probe, *IGH/BCL2* fusion probe
Moench et al., 2016 [42]	1 (33.3%)	2 (66.7%)	3	7 (87.5%)	1 (12.5%)	8	N/A	N/A	N/A	*MYC*, *BCL2*, *BCL6* break apart probes, *IGH/MYC* fusion probe, *IGH/BCL2* fusion probe
Miyaoka et al., 2018 [44]	3 (30.0%)	7 (70.0%)	10	N/A	N/A	N/A	N/A	N/A	N/A	*MYC*, *BCL2*, *BCL6* break apart probes, *IGH/MYC* fusion probe
Liu et al., 2022 [54]	6 (85.7%)	1 (14.3%)	7	3 (100.0%)	0 (0.0%)	3	0 (0.0%)	1 (100.0%)	1	*MYC*, *BCL2*, *BCL6*, *IGH* break apart probes, *IGH/MYC* fusion probe, *IGH/BCL2* fusion probe

Table S9: EBV positivity in DHL patients.

Study	Detection method	Positive (%)
Gebauer et al., 2013 [36]	IHC	0/7 (0.0)
Moench et al., 2016 [42]	IHC	0/6 (0.0)
Zhang et al., 2020 [47]	IHC	3/45 (6.7)
Ma et al., 2020 [48]	ISH	0/14 (0.0)
Tsai et al., 2021 [50]	ISH	0/20 (0.0)
Rodrigues-Fernandes et al., 2021 [49]	ISH	0/5 (0.0)
Liu et al., 2022* [54]	ISH	14/896 (15.6%)

*Study included a mixed cohort of DHL and THL

Figure S1: Funnel plots.

DHL proportions

Ann Arbor Stage 3-4

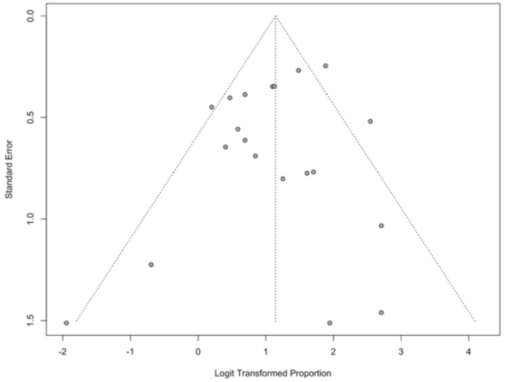

IPI 3-5

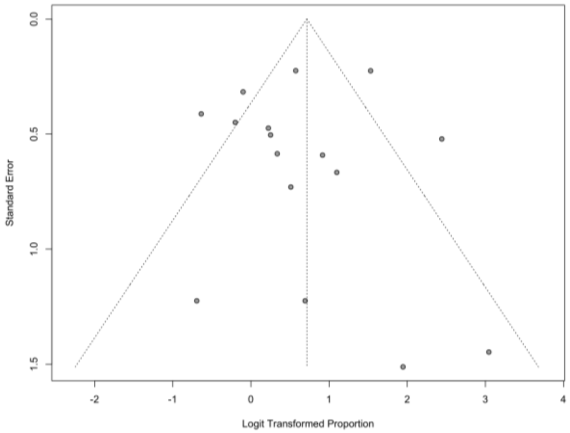

Elevated LDH

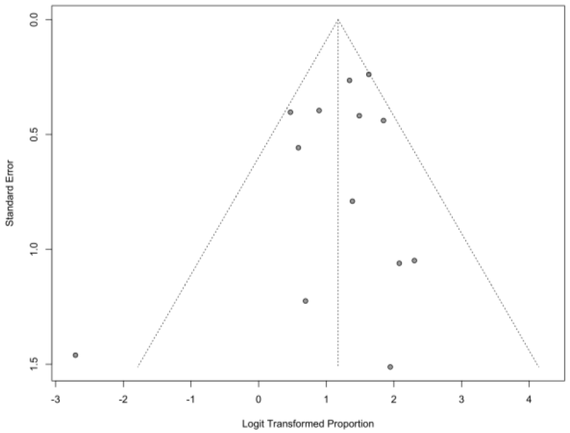

Presence of extranodal involvement

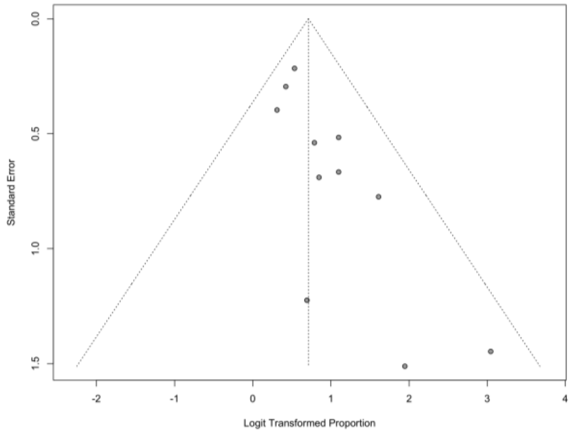

Bone marrow involvement

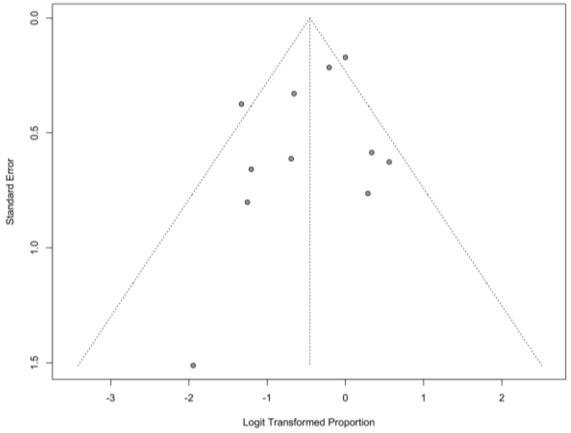

CNS involvement

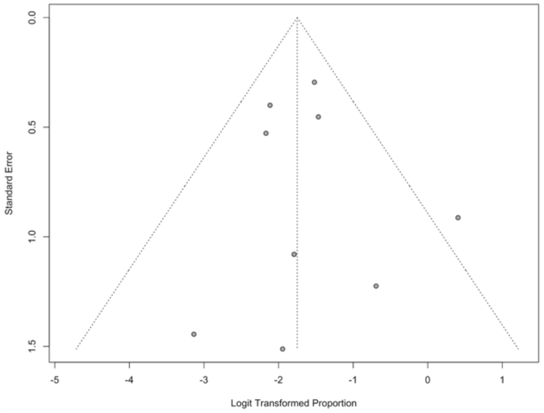

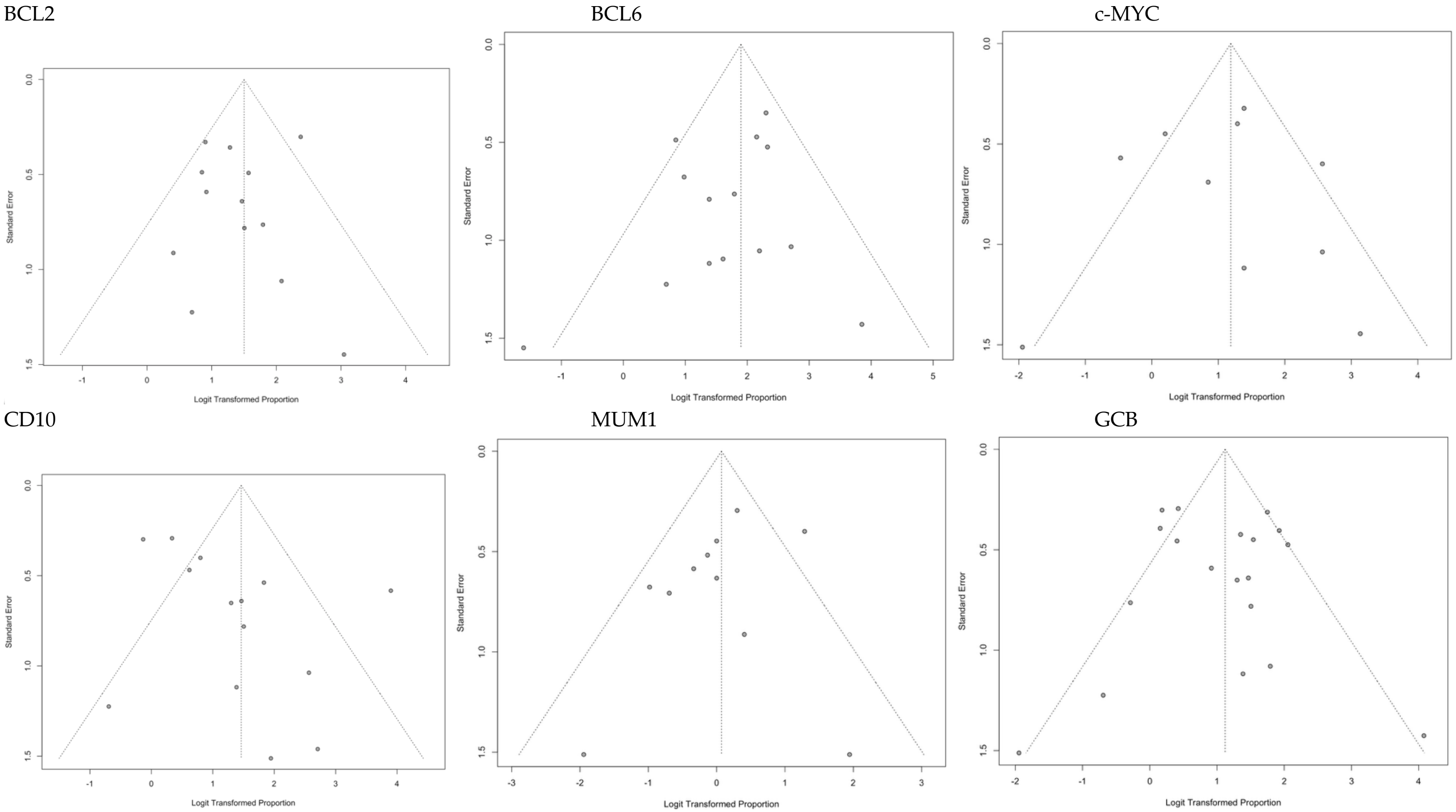
BCL2
BCL6
c-MYC
CD10
MUM1
GCB
Standard Error
Logit Transformed Proportion

MYC/BCL2 DHL proportions

Ann Arbor Stage 3-4

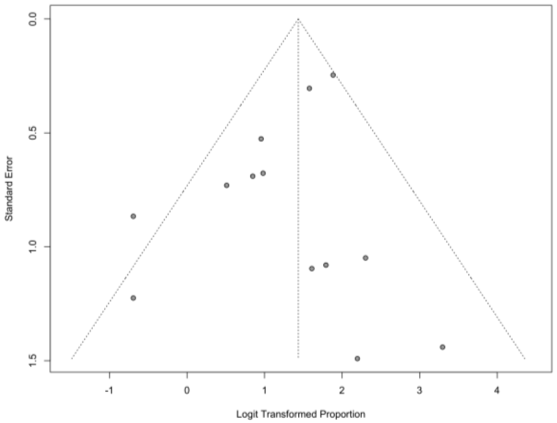

IPI 3-5

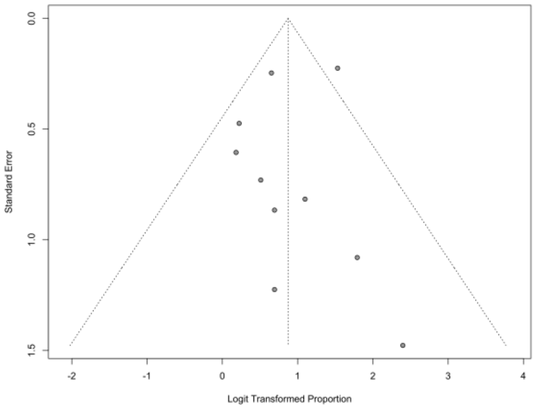

BCL2

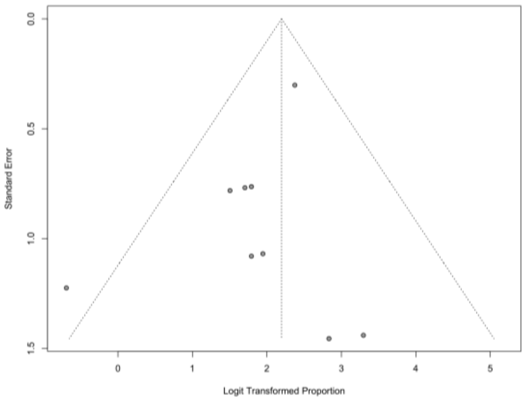

BCL6

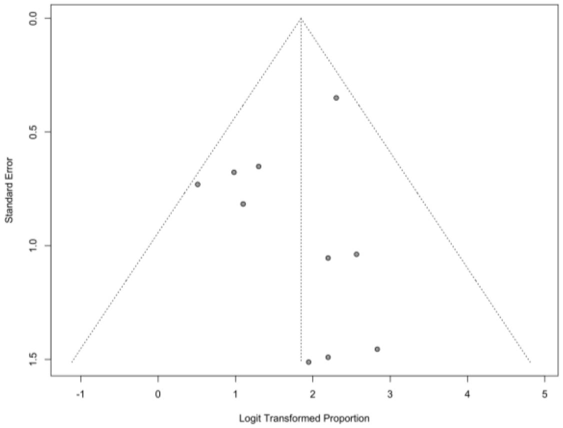

CD10

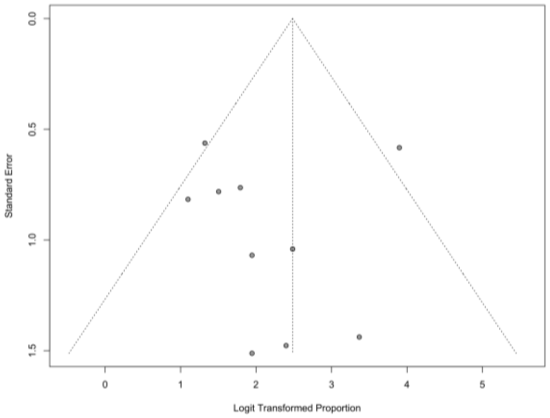

GCB

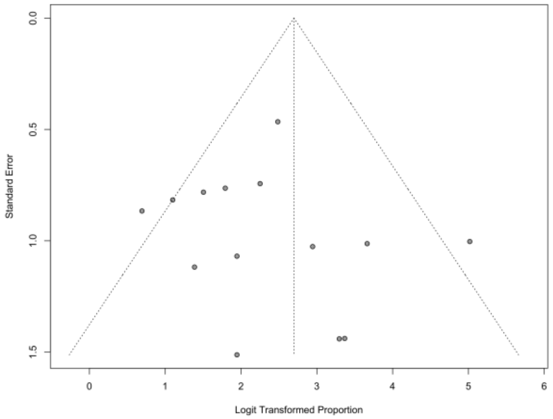

<u>*MYC/BCL6* DHL proportions</u>

Ann Arbor Stage 3-4

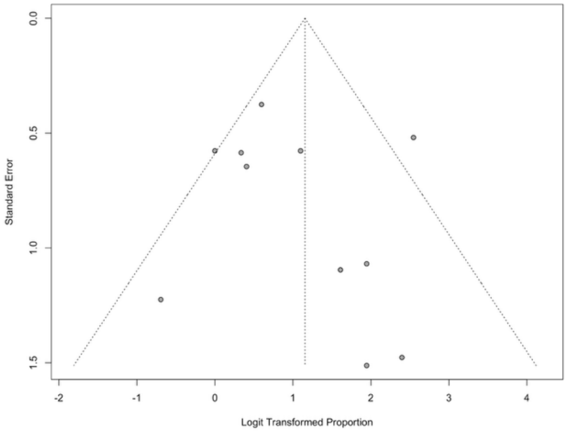

GCB

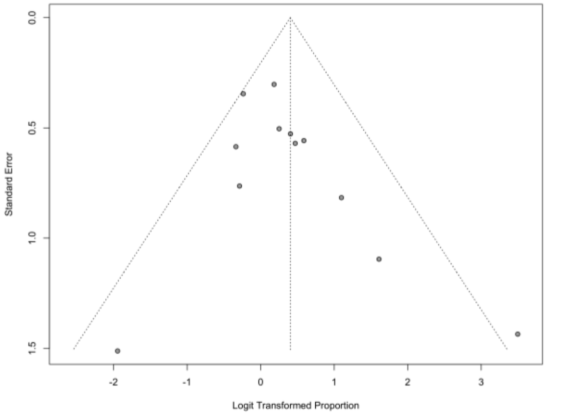

Supplement: Supplementary file 1 [file biomedicines-14-01375-s001.zip › biomedicines-4308624-supplementary.pdf]
